# Thiophene π-Bridge Engineering for Boosting Photocatalytic H_2_ Evolution of Dioxythiophene-Based D-A-π-A Conjugated Polymers Without Extraneous Noble Metal Loading

**DOI:** 10.3390/molecules31173115

**Published:** 2026-09-05

**Authors:** Guangsen Tian, Hongxi Zhao, Jinchen Zhang, Shaojia Song, Linfeng Zhang, Huadong Wu, Feng Wang, Jianding Li, Jia Guo, Qun Yi

**Affiliations:** 1 State Key Laboratory of Green and Efficient Development of Phosphorus Resources, Key Laboratory for Green Chemical Process of Ministry of Education, Hubei Key Laboratory of Novel Reactor and Green Chemical Technology, Engineering Research Center of Phosphorus Resources Development and Utilization of Ministry of Education, School of Chemical Engineering and Pharmacy, Wuhan Institute of Technology, Wuhan 430205, China; 15228972673@163.com (G.T.);; 2 College of Chemistry and Chemical Engineering, Taiyuan University of Technology, Taiyuan 030024, China; 3 School of Chemical Engineering and Technology, Tianjin University, Tianjin 300072, China; 4 School of Physical Education, Wuhan Business University, Wuhan 430056, China; 5 School of New Energy and Materials, Huzhou Normal University, Huzhou 313000, China

**Keywords:** 3,4-ethylenedioxythiophene, dibenzothiophene sulfone, donor–acceptor conjugated polymers, photocatalytic hydrogen evolution

## Abstract

Conjugated polymers featuring donor–acceptor (D-A) architectures have emerged as promising candidates for visible-light-driven hydrogen evolution, owing to their tunable optoelectronic properties. However, achieving high photocatalytic activity without noble-metal cocatalysts remains challenging. Herein, we report a series of D-A type conjugated polymers based on dibenzothiophene sulfone (BTDO) as an electron acceptor and 3,4-ethylenedioxythiophene (EDOT) as an electron donor, synthesized via Suzuki polycondensation. By optimizing the donor/acceptor feed ratio, the optimal copolymer, EDOT-BTDO-5, delivers a hydrogen evolution rate (HER) as high as 87.5 mmol h^−1^ g^−1^ was achieved under visible-light irradiation (λ > 420 nm) without any Pt cocatalyst. To further boost the charge separation efficiency, a thiophene π-bridge was introduced, yielding a D-A-π-A ternary copolymer, EDOT-BTDO-T, which exhibits a significantly enhanced HER of 103.45 mmol h^−1^ g^−1^, along with remarkable operational stability, retaining ~69% of its initial activity after 20 h of continuous illumination. Comprehensive characterization, including photoelectrochemical analysis and density functional theory (DFT) calculations, reveals that the incorporation of EDOT broadens the visible-light absorption range, while the thiophene π-bridge extends π-conjugation, and facilitates efficiency. This work demonstrates a molecular engineering strategy to construct high-performance, metal-free organic photocatalysts by tailoring D-A and D-A-π-A architectures, providing valuable insights for sustainable photochemical energy conversion.

## 1. Introduction

The ever-increasing global energy demand, coupled with the environmental consequences of fossil fuel combustion, necessitates the urgent advancement in clean and renewable energy technologies. Hydrogen, with its superior energy density and zero-carbon emissions upon combustion, stands out as an ideal energy carrier. Among various hydrogen production methods, photocatalytic water splitting using solar energy represents a particularly attractive route, enabling the direct conversion of abundant solar energy into storable chemical fuel [1,2,3,4,5]. Since the seminal work by Fujishima and Honda on TiO_2_ photoelectrodes [6], substantial efforts have been devoted to developing efficient photocatalysts. While inorganic semiconductors such as TiO_2_, CdS, and g-C_3_N_4_ have been extensively investigated, they often suffer from drawbacks including limited visible-light absorption, high cost, and potential toxicity [7,8,9,10].

In recent years, organic semiconductor photocatalysts have garnered immense interest due to their structural diversity, synthetic tunability, and the ability to finely modulate their electronic and optical properties through molecular design [11,12,13,14,15]. Among these, conjugated polymers featuring D-A frameworks have proven particularly effective. The periodic arrangement of electron-donating and electron-withdrawing moieties gives rise to an intramolecular push-pull interaction, which narrows the optical bandgap, enhances visible-light harvesting, and facilitates intramolecular charge transfer, thereby inhibiting the recombination of photogenerated charge carriers [16,17,18,19,20]. Further optimization of D-A systems involves the introduction of a π-bridge between the donor and acceptor, forming a D-π-A configuration. The π-bridge extends the effective conjugation length, improves molecular planarity, and provides a more efficient pathway for charge carrier transport, leading to superior photocatalytic performance [21,22,23,24,25,26,27].

Dibenzothiophene sulfone (BTDO) has emerged as a robust electron-accepting building block for constructing high-performance photocatalysts. Its planar, rigid structure and the strong electron-withdrawing sulfonyl group contribute to excellent chemical stability, enhanced hydrophilicity, and suitable energy levels for proton reduction [28,29,30]. For instance, poly (dibenzothiophene sulfone) (PDBTSO) itself shows photocatalytic activity, yet its charge transport efficiency is limited due to the lack of an internal donor moiety [31]. To overcome this limitation, incorporating appropriate electron donors to form D-A or D-π-A structures is a promising strategy. Thiophene-based donors, such as thieno 3,2-bthiophene, have been successfully employed for this purpose [31,32]. In parallel, 3,4-ethylenedioxythiophene (EDOT) is a particularly appealing donor unit. The fused structure of EDOT, featuring oxygen atoms in the dioxane ring, provides strong electron-donating ability, excellent π-conjugation, and good stability. The polar oxygen atoms can also enhance interaction with water, which is beneficial for surface reactions. The electronic structure of EDOT, with its high-lying HOMO level, makes it an ideal candidate for constructing efficient D-A systems with strong internal electric fields to drive charge separation [33,34,35].

In this study, we report a systematic molecular engineering approach to develop high-performance, metal-free photocatalysts based on BTDO and EDOT. First, a panel of D-A-type conjugated polymers (EDOT-BTDO-x) were synthesized by Suzuki polycondensation, and the optimal donor/acceptor ratio was determined. To further enhance charge separation and transport, a thiophene π-bridge was introduced between the EDOT donor and BTDO acceptor to construct a D-A-π-A ternary copolymer (EDOT-BTDO-T). The resulting photocatalysts were thoroughly characterized, and their photocatalytic H_2_ production performance was assessed under visible light. Through a combination of photoelectrochemical measurements and density functional theory (DFT) calculations, we elucidate the structure–property–activity relationships, demonstrating that the synergistic combination of EDOT donor and thiophene π-bridge significantly boosts the intrinsic activity, achieving remarkable hydrogen evolution rates without the need for any noble-metal cocatalyst.

## 2. Results and Discussion

### 2.1. Structural Characterization

**Figure 1:**
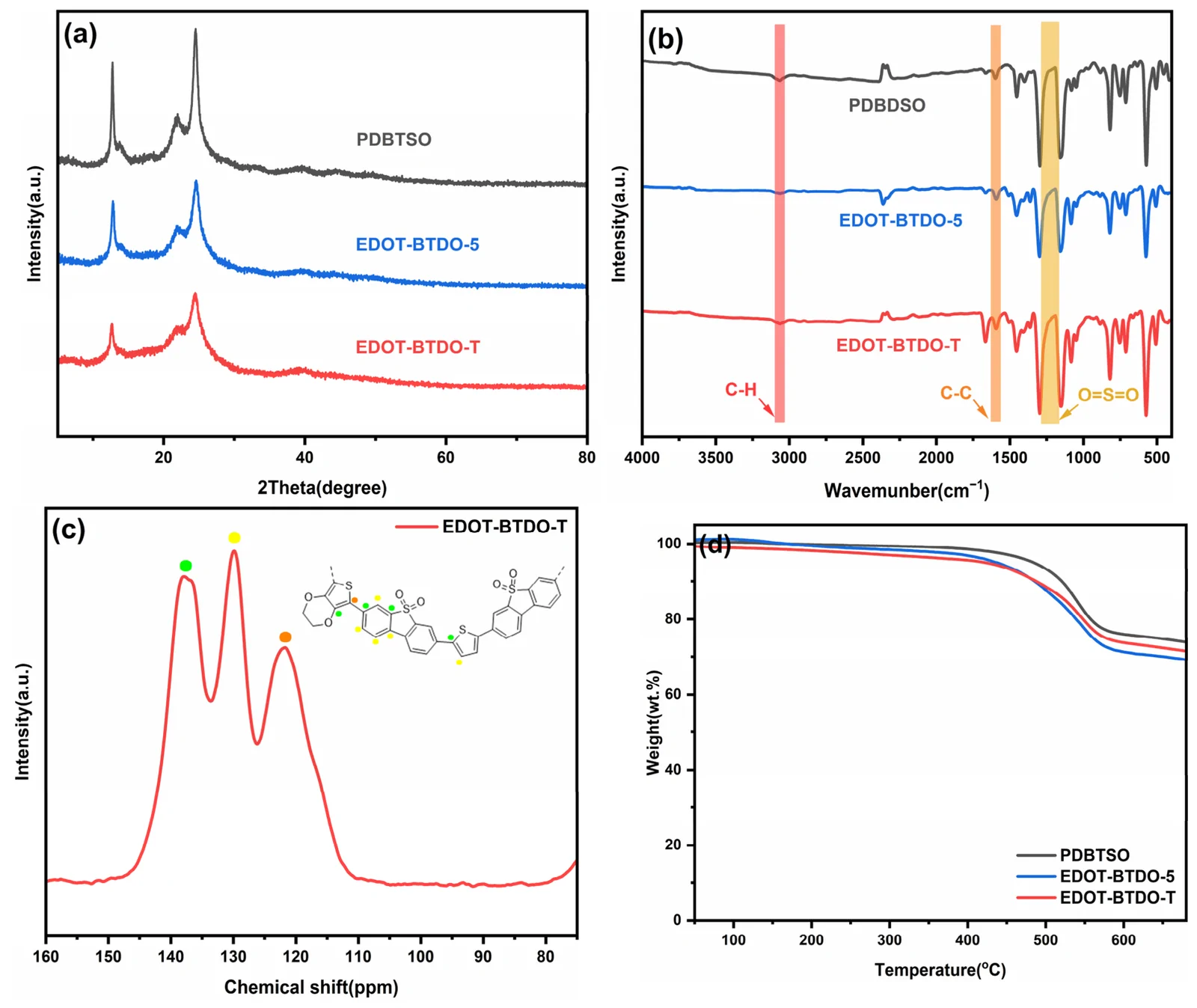
(**a**) XRD patterns of the copolymers, (**b**) FT-IR spectra of the copolymers, (**c**) CP-MAS NMR spectra of EDOT-BTDO-T, (**d**) TGA traces for the copolymers.

The structural characteristics of the synthesized polymers were first investigated by powder X-ray diffraction to assess their crystalline ordering and molecular packing. As shown in Figure 1a, the homopolymer PDBTSO exhibits two broad diffraction peaks centered at approximately 13° and 24°, which are characteristic of amorphous or low-crystallinity conjugated polymers. These peaks are typically assigned to lamellar packing and intermolecular π–π interactions between the aromatic polymer backbones, respectively. The presence of these broad features indicates that PDBTSO possesses a certain degree of local order despite its overall amorphous nature. Upon copolymerization with EDOT to form the binary D-A polymer EDOT-BTDO-5, the intensity of these diffraction peaks decreases noticeably, suggesting that the incorporation of the more flexible EDOT unit disrupts the molecular packing and reduces the overall ordering of the polymer chains. This increased chain flexibility likely arises from the less rigid nature of the EDOT unit compared to the more planar and rigid BTDO unit. Interestingly, the ternary copolymer EDOT-BTDO-T, which incorporates an additional thiophene π-bridge, displays the broadest and weakest diffraction peaks among the three samples. This indicates that the incorporation of the thiophene π-bridge further enhances the structural disorder, leading to the most amorphous character. While this reduced crystallinity might be perceived as a disadvantage for charge transport in some material systems, it can be beneficial for photocatalytic applications [36]. The enhanced disorder helps prevent overly tight π-π stacking, which can otherwise lead to excessive exciton quenching and charge recombination. Instead, the more disordered packing may facilitate the exposure of active sites and promote the diffusion of reactants to the catalyst surface, ultimately contributing to improved photocatalytic performance.

FT-IR spectroscopy was used to characterize the functional groups of EDOT-BTDO-5 and EDOT-BTDO-T and to verify the successful integration of the target moieties. As shown in Figure 1b, all three samples exhibit a series of characteristic absorption bands that are indicative of the BTDO core structure. Strong bands are observed at approximately 1157 and 1258 cm^−1^, which correspond to the asymmetric and symmetric stretching vibrations of the sulfonyl (O=S=O) group within the BTDO acceptor unit. These bands serve as a fingerprint for the successful integration of the BTDO unit into the polymer backbone. Additionally, a band around 1600 cm^−1^ is assigned to the aromatic C=C stretching vibrations of the conjugated backbone. Crucially, the absence of any characteristic vibrational peak near 1360 cm^−1^, which would correspond to the borinate ester group (B-O) from the starting monomer, provides unambiguous evidence that the Suzuki coupling reaction proceeded to completion. This observation confirms that the 3,7-bis(4,4,5,5-tetramethyl-1,3,2-dioxaborolan-2-yl)dibenzob [d, b] dthiophene 5,5-dioxide monomer was fully consumed during the polymerization, validating the successful preparation of the target copolymers.

The molecular structure of the ternary copolymer EDOT-BTDO-T was further clarified by solid-state ^13^C CP-MAS NMR spectroscopy. The resulting spectrum, presented in Figure 1c, displays a series of intense resonance signals in the range of 110–150 ppm, which is the characteristic chemical shift region for aromatic and thiophenic sp^2^-hybridized carbon atoms within the conjugated polymer backbone. This region provides valuable structural information about the different carbon environments. The signal around 120 ppm can be attributed to the carbon atoms of the thiophene rings that serve as the linkage points between the EDOT donor unit and the BTDO acceptor unit. The most intense peak, centered at approximately 130 ppm, is assigned to the aromatic carbon atoms of the BTDO acceptor unit itself, reflecting its dominant presence in the polymer structure. A distinct shoulder or peak near 140 ppm holds particular diagnostic significance. This signal can be assigned to two specific types of carbon environments: first, the carbon atoms within the EDOT unit that are directly bonded to the dioxane ring, and second, the carbon atoms at the connection sites between the thiophene π-bridge and the BTDO acceptor unit. The presence and distinctness of this signal provide direct spectroscopic evidence for the formation of these crucial connecting sites, confirming that all three components—EDOT, BTDO, and the thiophene π-bridge—are successfully integrated into a single, covalently connected polymer backbone. The well-resolved nature of these signals also suggests that the polymer possesses a reasonably homogeneous chemical environment with a consistent connectivity pattern.

The thermal resistance of the synthesized polymers, a key parameter for their potential application in long-term photocatalytic reactions, was assessed by thermogravimetric analysis (TGA) under nitrogen. The TGA and DTG curves, presented in Figure 1d and Figure S1, reveal that all three synthesized polymers exhibit excellent thermostability, which is a desirable characteristic for photocatalysts that may experience localized heating under continuous illumination. From ambient temperature to approximately 200 °C, all samples show a negligible weight loss of around 1–2%, which can be ascribed to the evaporation of physically physisorbed water and residual organic solvents from the synthesis process. The onset of significant thermal decomposition occurs at temperatures exceeding 400 °C for all three polymers, with initial decomposition temperatures (T_d_) estimated to be around 450 °C. Between 200 °C and 500 °C, a gradual weight loss of approximately 5–10% is observed, likely corresponding to the decomposition of less thermally stable side chains and minor main-chain scission events. The most pronounced weight loss occurs in the 500–600 °C range, which is ascribed to the extensive degradation and fragmentation of the polymer backbone. At 700 °C, the residual masses are 67.8% for PDBTSO, 73.5% for EDOT-BTDO-5, and 74.7% for EDOT-BTDO-T. Notably, the two copolymers exhibit slightly higher residual masses than the homopolymer, suggesting that the introduction of the EDOT donor and the thiophene π-bridge may enhance the overall thermal robustness. This can be mainly attributed to the higher sulfur/oxygen content in the molecular structure, as sulfur- and oxygen-containing units are more prone to crosslinking and aromatization during pyrolysis, thereby enhancing the char yield. In addition, the non-covalent interactions between S and O within the EDOT units can reinforce interchain binding, further facilitating char formation upon thermal decomposition. Importantly, the decomposition temperatures well above 400 °C are more than sufficient for stable operation under typical photocatalytic reaction conditions, which are usually conducted at or near room temperature. This excellent thermal stability ensures that the structural stability of the polymers is kept throughout the catalytic process, preventing thermal degradation that could lead to deactivation.

The morphological features and surface elemental distribution of the ternary copolymer EDOT-BTDO-T were examined by scanning electron microscopy (SEM) coupled with energy-dispersive X-ray (EDX) mapping. The SEM images, shown in Figure S2, reveal that EDOT-BTDO-T adopts an irregular, aggregated morphology consisting of particles with dimensions of approximately 1–2 μm. The surface of these particles appears rough and features abundant porous structures and crevices. This rough and porous morphology is advantageous for photocatalytic applications, as it provides an elevated specific surface area that can accommodate a greater number of catalytically active sites. The porous structure also facilitates the migration of reactant molecules (such as water and protons) to the catalytic sites and the release of product hydrogen gas, potentially enhancing the overall reaction kinetics. EDX elemental mapping was performed to assess the spatial configuration of the constituent elements within the polymer. The mapping images demonstrate a remarkably homogeneous distribution of carbon (C), oxygen (O), and sulfur (S) throughout the entire observed sample region. Carbon, as the backbone element, is uniformly distributed, confirming the continuity of the conjugated polymer network. Oxygen, originating from the sulfonyl groups of BTDO and the ether oxygen atoms of EDOT, is evenly dispersed, indicating that the donor and acceptor units are well mixed at the molecular level without significant phase separation. Similarly, sulfur, present in all three building blocks (EDOT donor, thiophene π-bridge, and BTDO acceptor), shows a uniform distribution, further confirming the molecular-scale homogeneity of the ternary copolymer. This uniform elemental distribution is crucial for ensuring consistent optoelectronic properties throughout the copolymers and for preventing the formation of localized charge recombination centers that could arise from phase-separated domains.

The surface wettability of the polymers was evaluated through static water contact angle measurements, as the interaction between the catalyst surface and the aqueous reaction medium is a critical factor affecting photocatalytic activity. The results, presented in Figure S3, show that PDBTSO exhibits a water contact angle of 73.2°, indicating a moderately hydrophilic surface. Upon copolymerization with EDOT to form EDOT-BTDO-5, the contact angle decreases to 61.2°, representing a significant increase in surface hydrophilicity. This enhancement can be attributed to the introduction of the EDOT unit, which contains polar oxygen atoms within its dioxane ring structure. These polar groups enhance the polymer’s ability to interact with water molecules through hydrogen bonding, thereby reducing the water contact angle. The ternary copolymer EDOT-BTDO-T exhibits a very similar contact angle of 61.7°, suggesting that the additional thiophene π-bridge does not substantially alter the surface wettability. The improved hydrophilicity of the copolymers is beneficial for photocatalytic hydrogen evolution, as it promotes better dispersion of the catalyst particles in the aqueous reaction medium and facilitates the uptake of water molecules and protons onto the surface of the catalyst. This enhanced interfacial contact can lead to more efficient utilization of photogenerated charge carriers for surface redox reactions, which ultimately accounts for the superior photocatalytic activity of these materials.

**Figure 2:**
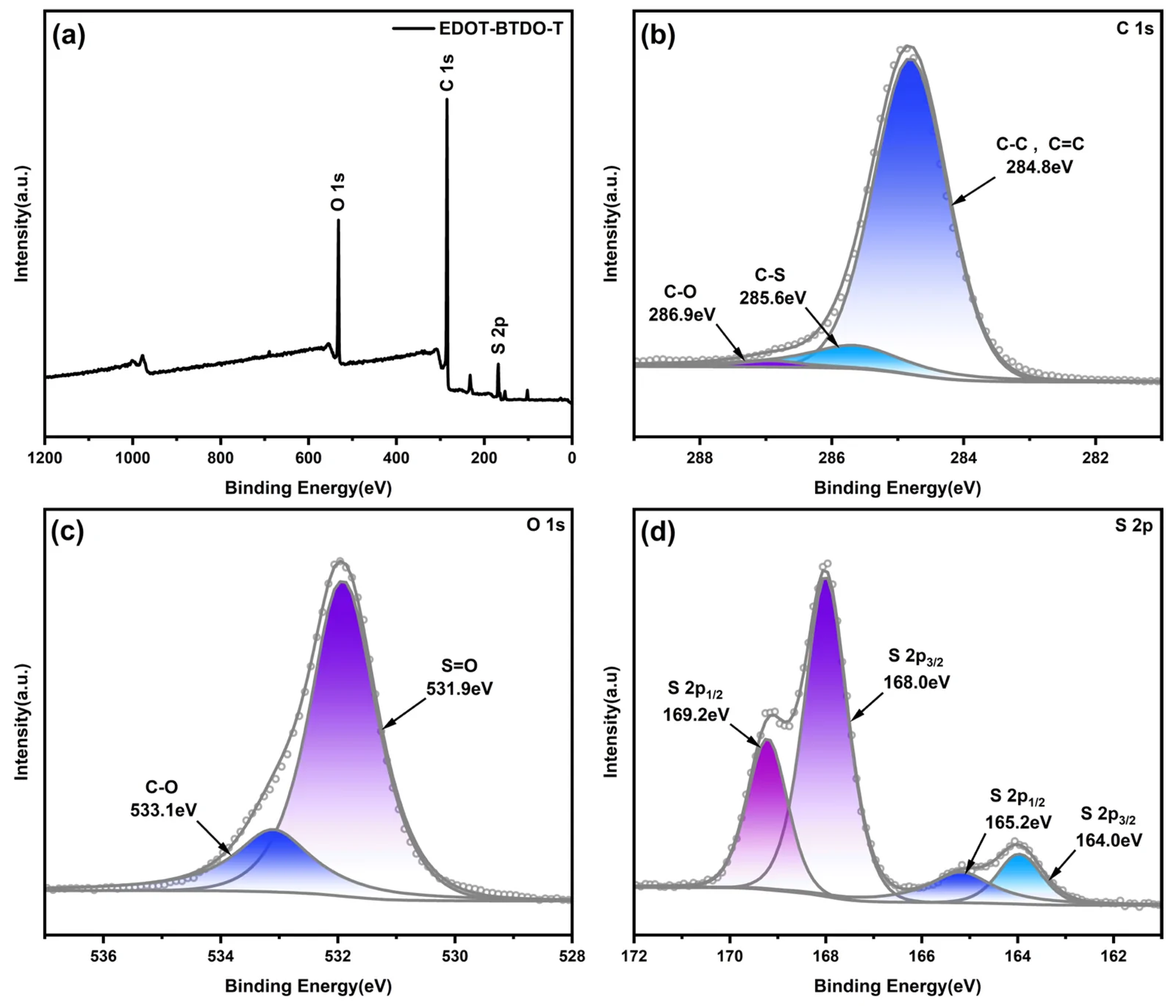
(**a**) XPS survey spectrum of EDOT-BTDO-T; High resolution XPS spectrum of EDOT-BTDO-5; (**b**) C 1s; (**c**) S 2p and (**d**) O 1s.

X-ray photoelectron spectroscopy (XPS) was utilized to conduct a detailed analysis of the chemical composition and bonding configurations on the surface of the ternary copolymer EDOT-BTDO-T. Comparative measurements were also carried out on the copolymers of PDBTSO and EDOT-BTDO-5 to track the structural evolution along the series, as shown in the XPS spectra in Figure 2 and Figure S4. The survey spectrum of EDOT-BTDO-T, presented in Figure 2a, clearly displays characteristic peaks corresponding to carbon (C 1s at ca. 284 eV), oxygen (O 1s at ca. 532 eV), and sulfur (S 2p in the range of 164–169 eV). The presence of these three elements is in good agreement with the theoretical elemental composition of the polymer backbone and confirms that no significant impurities are present on the surface. The high-resolution C 1s spectrum of EDOT-BTDO-T, presented in Figure 2b, can be fitted into three distinct peaks. The dominant peak at 284.6 eV is assigned to the C-C and C=C bonds of the aromatic rings and the conjugated backbone, representing the primary framework of the polymer. The peak at 285.6 eV corresponds to carbon atoms bonded to sulfur or oxygen (C-S or C-O), originating from the thiophene rings in the EDOT donor and the thiophene π-bridge. The peak at 286.9 eV is ascribed to carbon atoms bonded to oxygen in the C-O-C environment, which is characteristic of the ether oxygen linkages within the EDOT unit. The evolution of these peaks across the series provides evidence for the successful integration of the EDOT unit. The high-resolution O 1s spectrum of EDOT-BTDO-T, shown in Figure 2c, can be fitted with two peaks. The peak at 531.9 eV is assigned to the oxygen atoms of the sulfonyl (S=O) groups within the BTDO acceptor unit, serving as a clear signature of this electron-withdrawing component. The peak at 533.1 eV is ascribed to the oxygen atoms in the C-O-C ether linkages of the EDOT donor unit. The simultaneous presence of these two distinct oxygen environments provides direct evidence that both the BTDO acceptor and the EDOT donor are successfully integrated into the ternary copolymer structure.

The high-resolution S 2p core-level spectrum of EDOT-BTDO-T, presented in Figure 2d, provides the most definitive evidence for the successful fabrication of the D-A-π-A architecture. The spectrum can be fitted into two well-separated doublets. The doublet at higher binding energies, with peaks at 169.2 eV (S 2p_1/2_) and 168.0 eV (S 2p_3/2_), corresponds to the fully oxidized sulfur atoms present in the sulfone (SO_2_) groups of the BTDO acceptor unit. This characteristic doublet is present in all samples containing BTDO. The doublet at lower binding energies, with peaks at 165.2 eV (S 2p_1/2_) and 164.0 eV (S 2p_3/2_), is assigned to the sulfur atoms on the thiophene rings of the EDOT donor and the thiophene π-bridge. This doublet is absent in the spectrum of the homopolymer PDBTSO but appears prominently in both EDOT-BTDO-5 and EDOT-BTDO-T, confirming the successful integration of the thiophene-based units. In the same spectrum, the distinct separation and definitive presence of these two sulfur chemical environments undoubtedly verify the coexistence of EDOT donor units and BTDO acceptor units in covalently bonded polymer chains, whereas the bonding configuration between them remains inconclusive. The clear separation and unambiguous presence of these two sulfur environments within the same spectrum unequivocally confirm the coexistence of the EDOT donor unit (along with the thiophene π-bridge) and the BTDO acceptor unit within a single covalently linked polymer chain. This structural feature is the foundation for the efficient intramolecular charge transfer that underlies the superior photocatalytic performance of these materials.

### 2.2. Photoelectric Chemical Test

**Figure 3:**
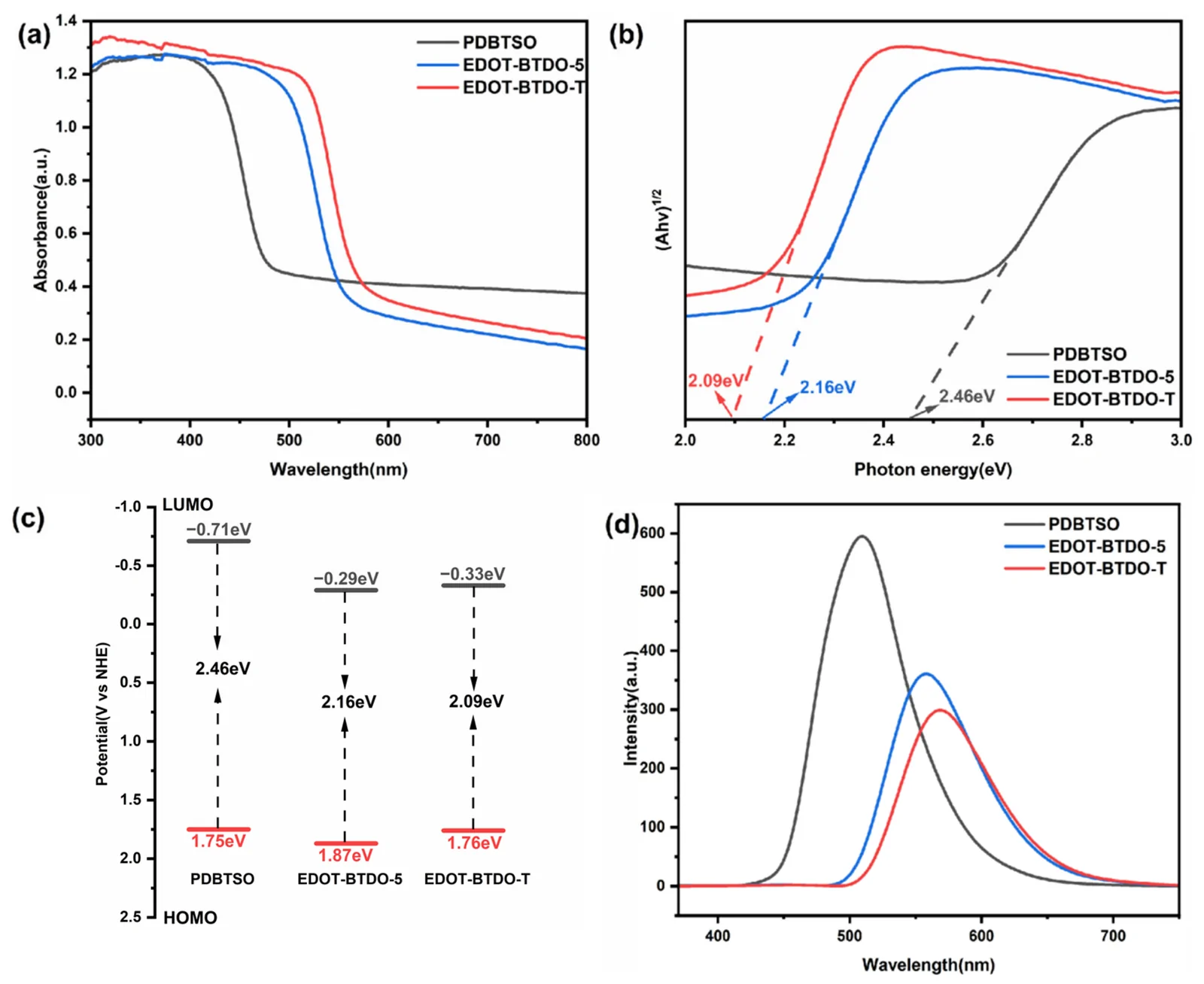
(**a**) Uv-Vis absorption spectra of PDBTSO, EDOT-BTDO-5 and EDOT-BTDO-T, (**b**) Tauc plots, (**c**) Energy band gap positions of the three polymers and (**d**) PL spectra of the three polymers.

The optical behavior of the synthesized polymers was comprehensively investigated using ultraviolet-visible (Uv-Vis) diffuse reflectance spectroscopy to evaluate their light-harvesting capabilities. The Uv-Vis absorption spectra, presented in Figure 3a, reveal that all three polymers exhibit broad absorption bands extending from the ultraviolet region well into the visible spectrum (300–600 nm), which is a characteristic feature of extended π-conjugated systems. This broad absorption is crucial for efficient solar energy utilization under visible-light irradiation. Compared to the homopolymer PDBTSO, both copolymers show a significant red shift in their absorption onset, with the ternary copolymer EDOT-BTDO-T displaying the most pronounced shift toward longer wavelengths. This red shift indicates a narrowing of the optical bandgap and an enhancement of visible-light absorption capability. The enhanced absorption can be attributed to two factors: first, the formation of the D-A structure creates an internal push-pull effect that reduces the bandgap, and second, the introduction of the thiophene π-bridge further extends the effective conjugation length of the polymer backbone, lowering the energy required for electronic transitions [16]. The optical bandgaps (E_g_) were quantitatively determined from Tauc plots, as shown in Figure 3b. The calculated values are 2.46 eV for PDBTSO, 2.16 eV for EDOT-BTDO-5, and 2.09 eV for EDOT-BTDO-T. The progressive narrowing of the bandgap from the homopolymer to the binary copolymer and further to the ternary copolymer confirms the effectiveness of the D-A and D-A-π-A molecular design strategies in tuning the electronic structure to achieve better visible-light response.

The energy band positions of the polymers were determined through an integration of Mott-Schottky analysis and XPS valence band spectroscopy. Mott-Schottky measurements were performed at multiple frequencies, and the resulting plots are shown in Figure S5. The positive slopes of the Mott-Schottky plots confirm that all three polymers exhibit n-type semiconducting behavior, which is typical for organic semiconductors with electron-rich donors. The flat-band potentials (E_fb_), which approximate the conduction band (CB) positions for n-type semiconductors, were determined from the intercept of the linear region of the plots [37]. The E_fb_ values are −0.71 V, −0.29 V, and −0.33 V (vs. NHE) for PDBTSO, EDOT-BTDO-5, and EDOT-BTDO-T, respectively. XPS valence band spectra, shown in Figure S6, were used to directly measure the valence band (VB) positions. The VB edges are 1.75 eV, 1.87 eV, and 1.76 eV (vs. NHE) for PDBTSO, EDOT-BTDO-5, and EDOT-BTDO-T, respectively. The consistency between the bandgaps derived from these electrochemical and optical methods validates the accuracy of the measurements. The complete band structures are schematically illustrated in Figure 3c. Critically, the conduction band positions of all three polymers are more negative than the standard reduction potential of H^+^/H_2_ (0 V vs. NHE), indicating that the photogenerated electrons are thermodynamically favorable for proton reduction to H_2_. This favorable energy level alignment is a fundamental prerequisite for photocatalytic hydrogen evolution.

Photoluminescence (PL) emission spectroscopy was used to investigate the recombination behavior of photogenerated electron-hole pairs, as the PL intensity is directly correlated with the efficiency of radiative recombination. The PL emission spectra were collected in the wavelength range of 350–800 nm with excitation at the maximum absorption wavelength of each sample, and the results are presented in Figure 3d. All three polymers exhibit characteristic emission bands in the 500–600 nm region, corresponding to the radiative relaxation of photogenerated excitons. However, the intensities of these emission bands vary dramatically across the series. PDBTSO exhibits the highest PL intensity, indicating that a large fraction of photogenerated electron-hole pairs undergo radiative recombination before they can participate in surface reactions. This behavior is typical for materials lacking an internal driving force for charge separation. For the binary copolymer EDOT-BTDO-5, the PL intensity is significantly reduced compared to PDBTSO. This quenching effect is a direct result of the D-A structure, where the repeated electron-rich and electron-deficient units create an internal electric field that promotes the separation of photogenerated electrons and holes [38]. This separation reduces the probability of their radiative recombination, leading to lower PL emission. Most notably, the ternary copolymer EDOT-BTDO-T exhibits the lowest PL intensity among the three samples. This further reduction in emission indicates that the introduction of the thiophene π-bridge within the D-A framework creates an even more efficient pathway for intramolecular charge transfer, allowing the photogenerated excitons to dissociate more rapidly and completely. The extended conjugation provided by the π-bridge enhances electronic coupling between the donor and acceptor [22,39], facilitating charge separation and effectively suppressing radiative recombination, thereby making more charge carriers available for the catalytic process.

To gain deeper insight into the dynamics of charge carrier separation and transport, time-resolved photoluminescence decay (TRPL) measurements were conducted. The fluorescence decay curves, presented in Figure S7, were fitted using a biexponential decay model to extract the average fluorescence lifetimes (τ_ave_), which provide quantitative information about the exciton recombination kinetics. The calculated τ_ave_ values are 1.72 ns for PDBTSO, 1.83 ns for EDOT-BTDO-5, and 2.02 ns for EDOT-BTDO-T. The homopolymer PDBTSO exhibits the shortest average lifetime, indicating that excitons recombine rapidly, consistent with its high PL intensity and low charge separation efficiency. The binary copolymer EDOT-BTDO-5 shows a modestly extended lifetime, suggesting that the D-A structure provides some degree of charge separation, delaying recombination. Remarkably, the ternary copolymer EDOT-BTDO-T exhibits the longest average fluorescence lifetime, representing a significant prolongation of the exciton lifetime. This extended lifetime is a direct consequence of the D-A-π-A architecture, where the thiophene π-bridge not only enhances the efficiency of exciton dissociation but also physically separates the photogenerated electron and hole across different molecular units. This spatial separation, combined with the improved charge transport pathway provided by the π-bridge, effectively suppresses geminate recombination and enables the separated charges to migrate to the catalyst surface where they are capable of participating in redox reactions. The prolonged charge carrier lifetime is a critical factor contributing to the outstanding photocatalytic activity of EDOT-BTDO-T.

**Figure 4:**
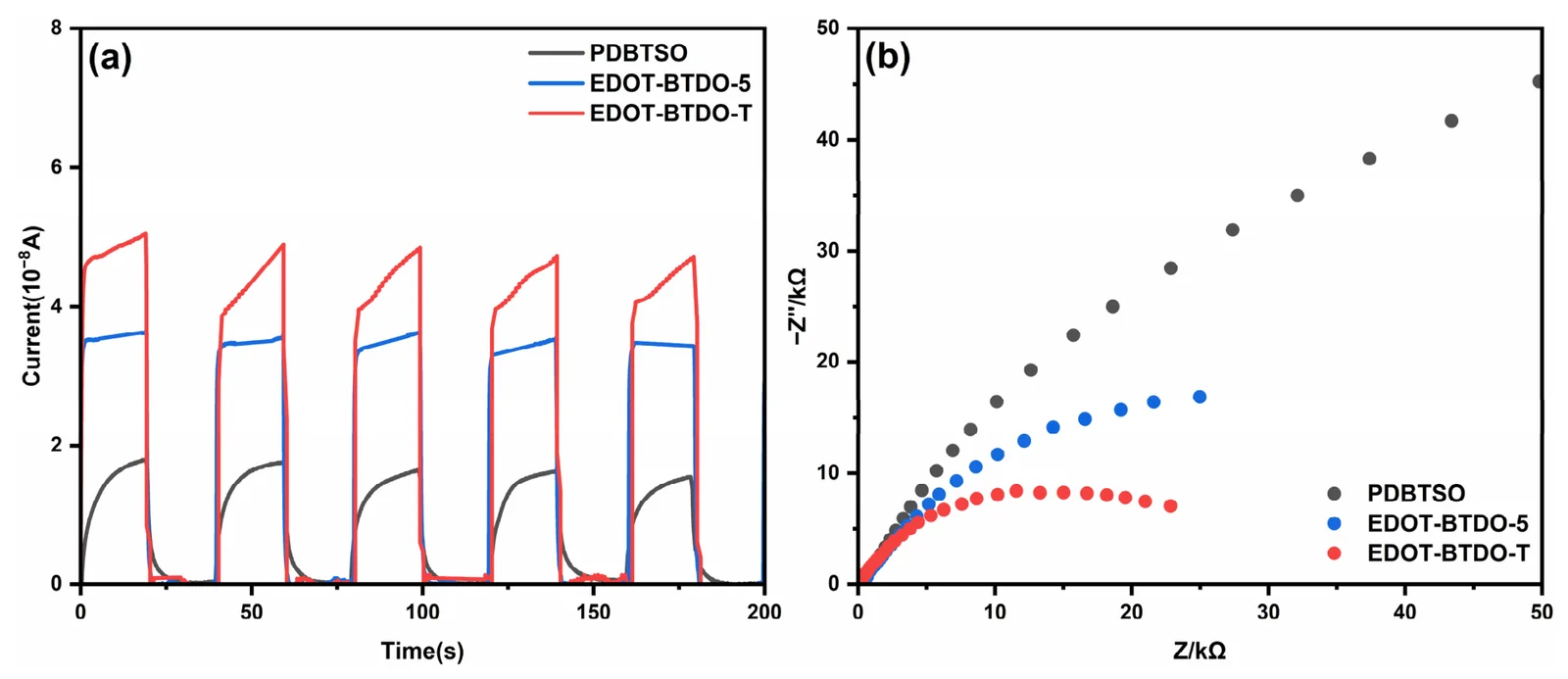
(**a**) Transient photocurrent and (**b**) EIS Nyquist plots of PDBTSO, EDOT-BTDO-5 and EDOT-BTDO-T.

The efficiency of photogenerated charge separation and transport was further assessed using transient photocurrent response and electrochemical impedance spectroscopy (EIS). Transient photocurrent measurements, presented in Figure 4a, were performed under intermittent visible-light illumination to evaluate the ability of the materials to generate and transport photocurrent. Upon illumination, all three polymers exhibit a rapid increase in photocurrent, followed by a rapid decay to a steady state when the light is turned off. PDBTSO generates the lowest photocurrent density, reflecting its poor charge separation and transport efficiency. EDOT-BTDO-5 shows a significantly higher photocurrent response, consistent with the improved charge separation enabled by the D-A structure. Notably, EDOT-BTDO-T exhibits the highest and most stable photocurrent density among the three samples. This enhanced photocurrent response indicates that the ternary copolymer not only generates more photogenerated charge carriers due to its broader light absorption but also achieves more efficient separation and transport of these carriers to the electrode surface. The enhanced charge transport performance is likely attributable to the extended conjugated structure and improved molecular planarity resulting from the introduction of the thiophene π-bridge.

Electrochemical impedance spectroscopy (EIS) was employed to investigate the charge transfer resistance at the interface between the electrode and the electrolyte. The Nyquist plots, presented in Figure 4b, reveal distinct differences in the semicircle diameters across the samples. In a Nyquist plot, the diameter of the high-frequency semicircle is equivalent to the charge transfer resistance (Rct), which reflects the ease with which photogenerated charge carriers can be transferred from the catalyst to the electrolyte. A reduced semicircle diameter corresponds to a lower charge transfer resistance and more efficient interfacial charge transfer. PDBTSO exhibits the largest semicircle diameter, indicating the highest charge transfer resistance and the most hindered interfacial charge transport. EDOT-BTDO-5 shows a significantly reduced semicircle diameter, indicating that the D-A structure facilitates charge transfer across the interface. Remarkably, EDOT-BTDO-T exhibits the smallest semicircle diameter, which corresponds to the lowest charge transfer resistance. This result provides direct evidence that the D-A-π-A architecture effectively reduces the energy barrier for charge transfer at the catalyst-electrolyte interface, allowing photogenerated electrons to be more readily transferred to the adsorbed reactants for hydrogen evolution. The combination of high photocurrent density and low charge transfer resistance confirms that EDOT-BTDO-T possesses optimal optoelectronic properties for efficient photocatalytic hydrogen production.

**Figure 5:**
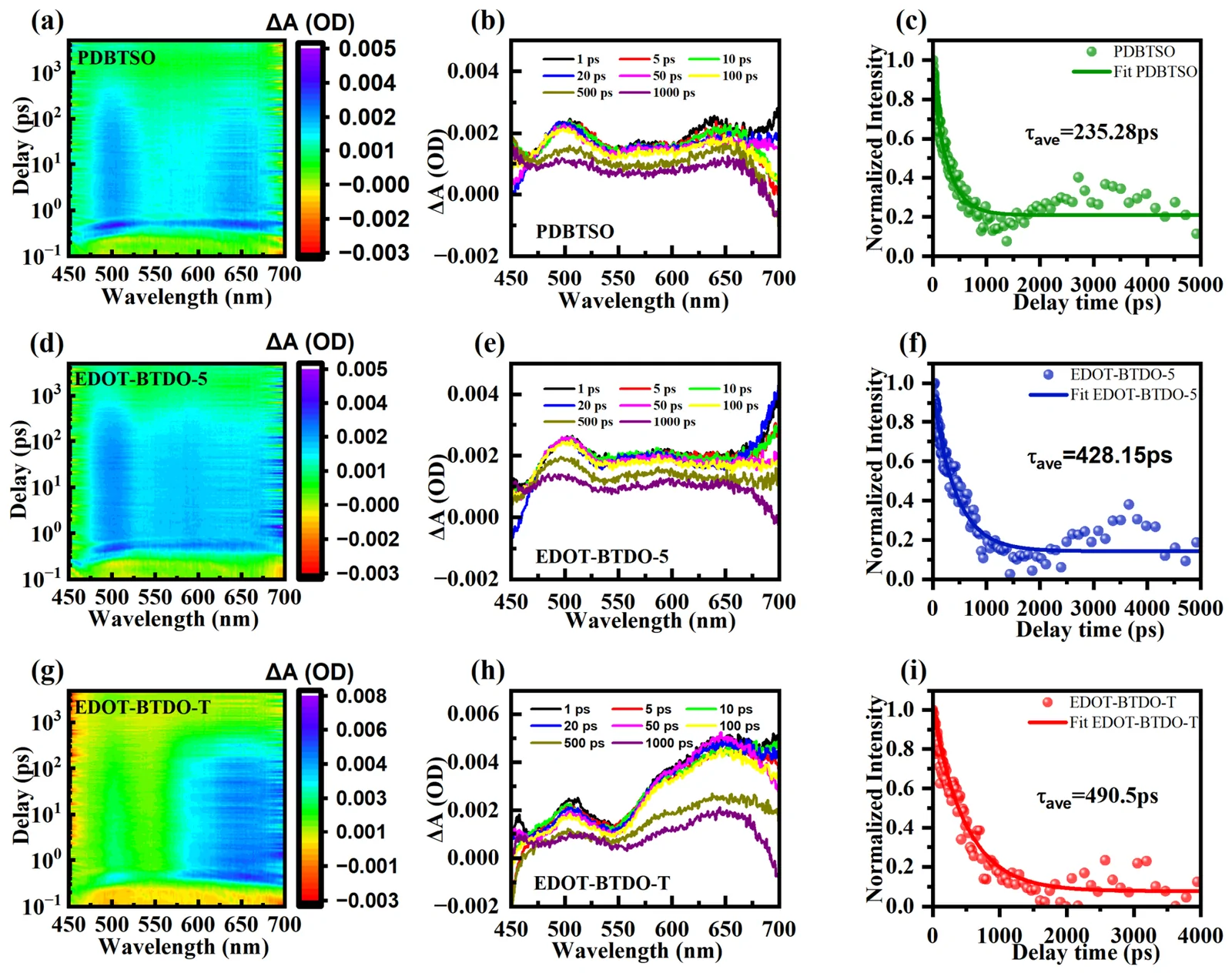
(**a**,**d**,**g**) fs-TAS spectra excited of PDBTSO, EDOT-BTDO-5 and EDOT-BTDO-T. (**b**,**e**,**h**) Corresponding spectra of PDBTSO, EDOT-BTDO-5 and EDOT-BTDO-T at selected pump–probe time delays. (**c**,**f**,**i**) Decay kinetic curves of PDBTSO, EDOT-BTDO-5 and EDOT-BTDO-T.

The exciton dynamics of PDBTSO, EDOT-BTDO-5, and EDOT-BTDO-T were investigated using femtosecond transient absorption (fs-TAS) spectroscopy, and the effects of subtle molecular structural modifications on photoelectric performance were examined (Figure 5). As shown in Figure 5b,e,h, upon photoexcitation, a pronounced and intense excited-state absorption (ESA) band was observed for EDOT-BTDO-T in the 600–700 nm region, accompanied by only a weak ground-state bleach (GSB) near 500 nm. In contrast, the GSB signals for PDBTSO and EDOT-BTDO-5 were predominantly centered around 500 nm, with much weaker ESA in the 600–700 nm range than that of EDOT-BTDO-T. These spectral differences indicate that the introduction of the thiophene unit (T) or alteration of the molecular framework significantly modulates the excited-state properties. The strong and red-shifted ESA observed in EDOT-BTDO-T is characteristic of long-lived charge-separated states, suggesting that photogenerated excitons undergo more efficient intra- or intermolecular charge transfer, thereby suppressing fast monomolecular recombination. By comparison (Figure 5c,f,i), PDBTSO is dominated by a rapidly decaying GSB with only weak ESA, implying that excitons primarily recombine monomolecularly without forming effective long-lived charge-separated states; EDOT-BTDO-5 exhibits intermediate behavior. The average lifetime (τ_ave_) of EDOT-BTDO-T reached 490.50 ps, markedly longer than that of EDOT-BTDO-5 (428.15 ps) and PDBTSO (235.28 ps). This macroscopically extended lifetime, particularly arising from its substantial long-lived component, can be attributed to more efficient charge-separation formation, which greatly suppresses photogenerated electron–hole recombination.

### 2.3. Photocatalytic Test

**Figure 6:**
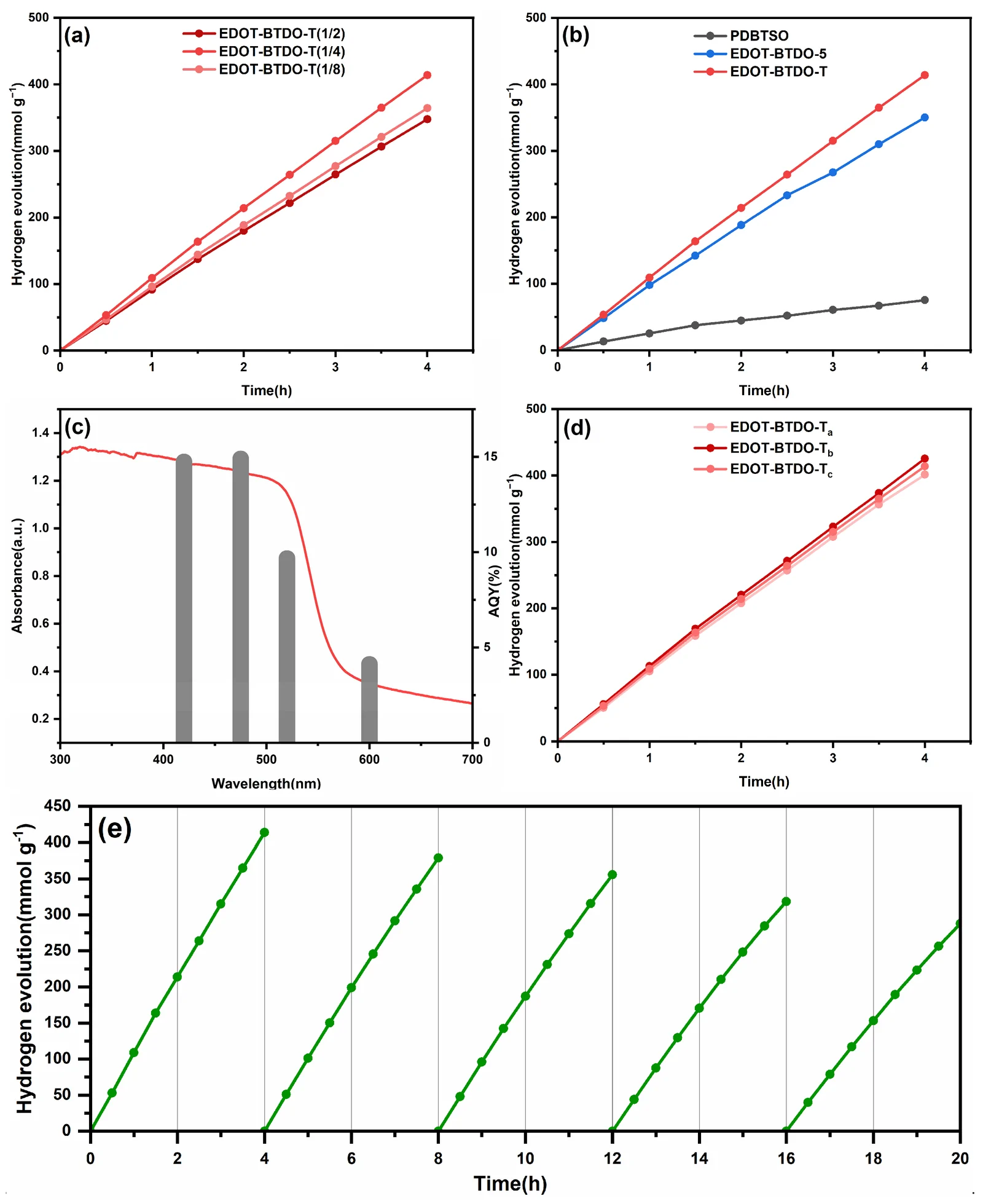
(**a**) Hydrogen evolution rate of EDOT-BTDO-T_x_, (**b**) Hydrogen evolution rate of PDBTSO, EDOT-BTDO-5 and EDOT-BTDO-T, (**c**) AQY of EDOT-BTDO-T, (**d**) Hydrogen production repeatability test of EDOT-BTDO-T and (**e**) 20 h photocatalytic cycling test of EDOT-BTDO-T.

The photocatalytic hydrogen evolution performance of the synthesized polymers was assessed under visible-light irradiation (λ > 420 nm) using ascorbic acid as a sacrificial donor and without any noble-metal cocatalyst. To first optimize the composition, a series of ternary copolymers denoted as EDOT-BTDO-Tx were synthesized by varying the feed molar fraction of the thiophene π-bridge monomer relative to the total thiophene and EDOT feed, specifically 1/2, 1/4, and 1/8 (denoted asT_1/2_, T_1/4_, and T_1/8_, respectively). As shown in Figure 6a, the hydrogen evolution rates of the three ternary copolymers are 87.54, 103.45, and 90.68 mmol h^−1^ g^−1^, respectively, all of which are slightly higher than that of the binary EDOT-BTDO-5, confirming the beneficial role of the π-bridge. However, the activity strongly depends on the amount of thiophene incorporated. The optimal performance is achieved for EDOT-BTDO-T_1/4_, while both excessive (T_1/2_) and insufficient (T_1/8_) π-bridge incorporation lead to lower activities. This volcano-shaped trend suggests that an appropriate π-bridge content is crucial for balancing extended conjugation and efficient charge transfer, without introducing excessive structural defects or charge trapping sites that could hinder carrier mobility.

The time-dependent hydrogen evolution profiles are presented in Figure 6b. All samples exhibit linear hydrogen evolution over the 4 h reaction period, indicating stable catalytic activity under the experimental conditions. PDBTSO, the homopolymer, shows a modest hydrogen evolution rate (HER) of 18.78 mmol h^−1^ g^−1^, which serves as a baseline for comparison. This activity, while detectable, is limited by the poor charge separation efficiency of the A-A type structure. The binary copolymer EDOT-BTDO-5 demonstrates a dramatically improved HER of 87.5 mmol h^−1^ g^−1^, representing an approximately 4.7-fold enhancement compared to PDBTSO. This substantial improvement unequivocally demonstrates the effectiveness of constructing a D-A structure in promoting photocatalytic activity. Moreover, the photocatalytic activity of the above-mentioned copolymer photocatalysts outperform the majority of the reported photocatalysts (Table 1). The incorporation of the electron-donating EDOT unit creates a strong internal push-pull effect that enhances light absorption, promotes charge separation, and facilitates charge transport, all of which contribute to the enhanced HER.

Most remarkably, the ternary copolymer EDOT-BTDO-T achieves an even higher HER of 103.45 mmol h^−1^ g^−1^, representing a 5.5-fold enhancement over PDBTSO and an 18% improvement over EDOT-BTDO-5. This further enhancement underscores the key role of the thiophene π-bridge in boosting the photocatalytic activity. The π-bridge serves multiple functions: it extends the effective conjugation length, enhancing visible-light absorption and narrowing the bandgap; it improves the molecular planarity, facilitating π-π stacking and charge transport; and it creates a more efficient pathway for intramolecular charge transfer from the donor to the acceptor. The synergistic combination of these effects results in the outstanding photocatalytic performance of EDOT-BTDO-T [55].

To evaluate the efficiency of photon-to-hydrogen conversion, the apparent quantum yield (AQY) of EDOT-BTDO-T was determined under monochromatic light irradiation at different wavelengths. The AQY values, presented in Figure 6c, are 14.75% at 420 nm, 14.91% at 475 nm, 9.68% at 520 nm, and 4.13% at 600 nm. The wavelength-dependent AQY trend closely follows the shape of the Uv-Vis absorption spectrum, with the highest AQY values coinciding with the region of strongest light absorption. This correlation confirms that the observed hydrogen evolution is indeed driven by photoexcitation of the polymer itself, rather than by any parasitic processes. The relatively high AQY values in the visible region demonstrate that EDOT-BTDO-T efficiently converts absorbed photons into hydrogen gas, highlighting the effectiveness of the D-A-π-A molecular design in optimizing the utilization of solar energy for photocatalytic H_2_ production.

Under identical conditions, three different batches of the catalyst were synthesized and subjected to 4 h hydrogen production tests. As shown in Figure 6d, the catalyst exhibited highly consistent hydrogen evolution rates across different batches, demonstrating the reproducibility of the polymer. In addition, a longer-term stability test was conducted over 20 h (five consecutive 4 h cycles), and the results are presented in Figure 6e. EDOT-BTDO-T retains approximately 69% of its initial activity after 20 h of continuous illumination, demonstrating good photostability and resistance to deactivation. This gradual activity decline is typical for organic photocatalysts and may be attributed to the slow accumulation of surface-adsorbed intermediates or minor structural relaxation, rather than catastrophic degradation of the polymer backbone.

To further elucidate the structural stability of the catalyst, FT-IR and solid-state ^13^C NMR analyses were performed on the used catalyst after the cycling test. The FT-IR spectra of the fresh and used catalysts, presented in Figure S8, show no discernible differences in the positions or relative intensities of the characteristic absorption bands. The sulfonyl group vibrations at 1157 and 1258 cm^−1^ remain unchanged, and the aromatic C=C stretching band at 1600 cm^−1^ is preserved. Similarly, the solid-state ^13^C NMR spectra of the fresh and used catalysts, presented in Figure S9, exhibit identical chemical shift patterns in the 110–150 ppm region, confirming that the aromatic and thiophenic carbon environments are intact. The absence of any new peaks or peak shifts indicates that no significant chemical degradation or structural rearrangement has occurred during the photocatalytic reaction. These results collectively demonstrate that EDOT-BTDO-T possesses excellent chemical and structural stability under the operating conditions, which is essential for its practical application in solar-driven H_2_ production.

Since palladium catalysts are indispensable for the Suzuki coupling reactions used in the synthesis of these polymers, the potential influence of residual palladium on the observed photocatalytic activity was carefully examined. Trace amounts of Pd could potentially act as cocatalysts for hydrogen evolution, artificially inflating the measured HER values. To address this concern, inductively coupled plasma optical emission spectroscopy (ICP-OES) was used to quantify the residual Pd content in the polymer samples. The analysis revealed Pd contents of 0.5 wt% for PDBTSO, 0.14 wt% for EDOT-BTDO-5, and 0.53 wt% for EDOT-BTDO-T, as shown in Figure S10. These values represent low, ppm-level concentrations that are typical for polymers synthesized via palladium-catalyzed cross-coupling reactions. Critically, the variation in Pd content across the three samples does not correlate with the observed trend in HER. PDBTSO, with a Pd content of 0.5 wt%, exhibits the lowest HER of 18.78 mmol h^−1^ g^−1^, while EDOT-BTDO-5, with the lowest Pd content of 0.14 wt%, shows a much higher HER of 87.5 mmol h^−1^ g^−1^. EDOT-BTDO-T, with the highest Pd content of 0.53 wt%, shows the highest HER of 103.45 mmol h^−1^ g^−1^. The lack of a consistent positive correlation between Pd content and HER strongly suggests that the residual palladium is not acting as a significant cocatalyst in these systems. Instead, the pronounced differences in photocatalytic performance are unequivocally attributed to the intrinsic differences in the molecular structures and optoelectronic properties of the polymers themselves, specifically the D-A and D-A-π-A architectures that govern charge separation and transport.

To gain a deeper understanding of the electronic structure and charge transfer mechanism on the molecular scale, density functional theory (DFT) calculations were performed on model fragments representing the D-A-π-A architecture of EDOT-BTDO-T. The optimized geometry of the model fragment is presented in Figures S11 and S12. The computed dihedral angles between the EDOT donor and the BTDO acceptor, and between the thiophene π-bridge and the BTDO acceptor, are significantly small, measuring approximately 18.8° and 27.0°, respectively. These small dihedral angles indicate a high degree of molecular planarity across the D-π-A unit. This near-planar conformation is highly beneficial for optoelectronic properties, as it maximizes the coupling of π-orbitals along the polymer backbone, thereby extending the effective conjugation length and facilitating efficient electronic communication between the donor and acceptor units. The planar structure also enhance charge transport in the solid state. The calculated frontier molecular orbitals, presented in Figures S13 and S14, reveal a distinct spatial separation of the HOMO and LUMO across the D-A-π-A framework. The HOMO (highest occupied molecular orbital) is predominantly localized on the EDOT donor unit, with significant electron density extending into the thiophene π-bridge. This indicates that the EDOT unit acts as the primary electron donor, while the π-bridge participates in the delocalization of the HOMO, facilitating hole transport [56,57]. In contrast, the LUMO (lowest unoccupied molecular orbital) is overwhelmingly concentrated on the BTDO acceptor unit, with some distribution extending onto the thiophene π-bridge. This clear spatial charge separation of the frontier orbitals is a direct manifestation of the D-A-π-A design. The energy offset between the HOMO of the donor and the LUMO of the acceptor, combined with the electronic coupling provided by the π-bridge, creates a strong driving force for intramolecular charge transfer. Upon photoexcitation, electrons are transferred from the EDOT donor to the BTDO acceptor, while the holes remain localized on the donor unit. This spatial separation of the photogenerated electron and hole significantly reduces the probability of geminate recombination, thereby extending the lifetime of the charge carriers and enhancing their availability for surface reactions. The electrostatic potential (ESP, Figure S15) map reveals strong negative potentials around the sulfone group and the oxygen atoms in the BTDO acceptor unit. In contrast, the EDOT donor unit exhibits an electron-deficient region. This uneven charge distribution clearly identifies electron-rich and electron-deficient sites. In this system, electrons and holes are concentrated on the acceptor and donor units, respectively. However, the ESP pattern shows significant polarization: the sulfone group acts as an electron trap (negative potential), whereas the EDOT unit serves as a hole trap (positive potential). This ESP-based spatial separation of the donor and acceptor is consistent with frontier orbital analysis, where the HOMO is concentrated on the donor and the LUMO on the acceptor. Moreover, the nearly planar D-A-π-A configuration creates a smooth potential gradient from the donor to the acceptor via the thiophene π-bridge, further enhancing the polarization. This charge distribution differs from that of traditional D-A molecules [58]. The strong localization of electrons and holes driven by ESP effectively suppresses exciton formation.

**Figure 7:**
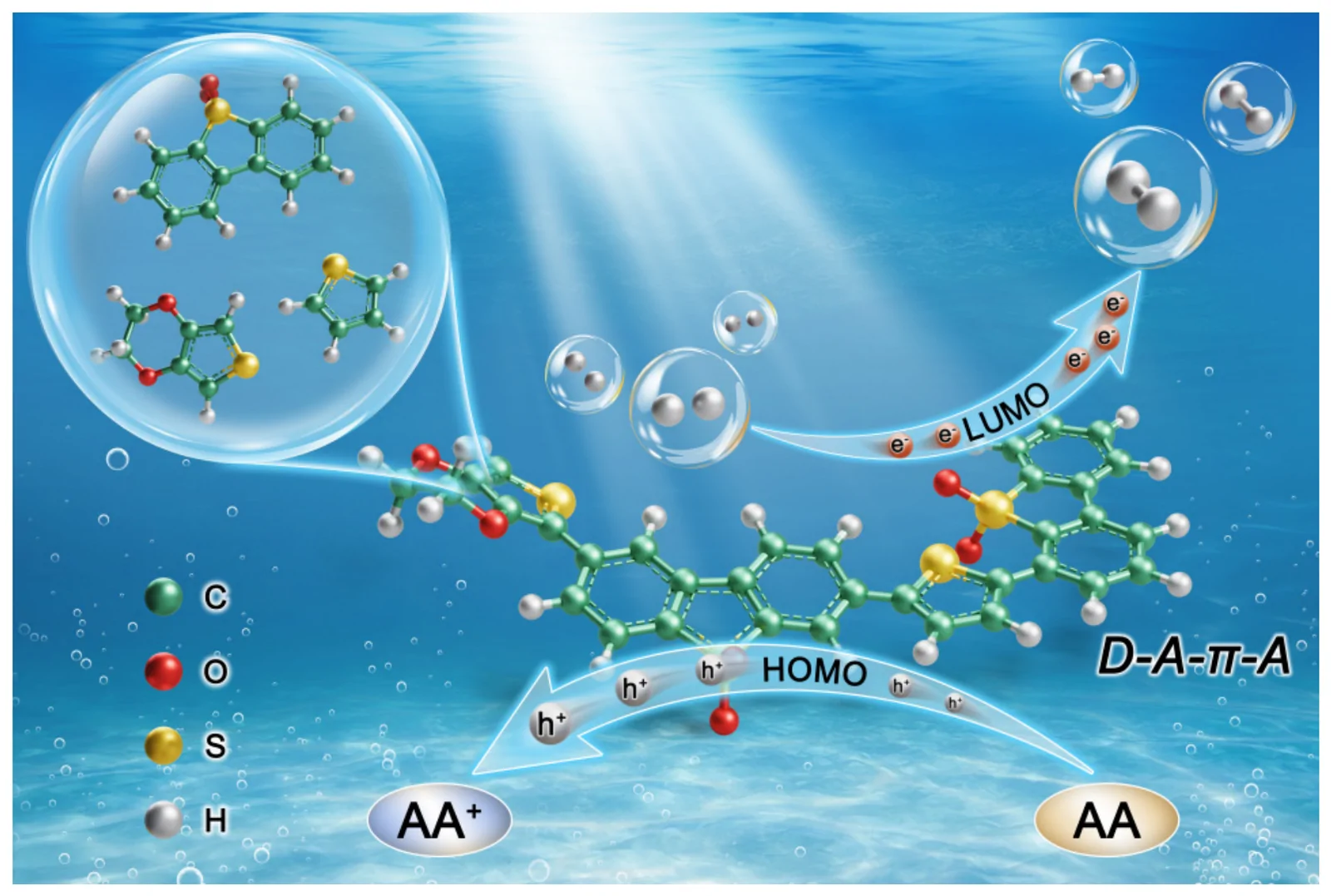
Schematic diagram of the photocatalytic mechanism of the D-A-π-A type structure.

Based on the comprehensive experimental and theoretical analyses, a proposed mechanism for the enhanced photocatalytic hydrogen evolution over the D-A-π-A copolymer EDOT-BTDO-T is schematically illustrated in Figure 7. Under visible-light irradiation, the polymer absorbs photons with energy equal to or greater than its bandgap, exciting electrons from the HOMO to the LUMO and generating bound excitons (electron-hole pairs). The strong intramolecular charge transfer effect, inherent to the D-A-π-A architecture, drives the rapid dissociation of these excitons. Specifically, the photogenerated electrons are efficiently transferred from the EDOT donor unit to the BTDO acceptor unit, and the thiophene π-bridge situated between them facilitates this process. This directional charge transfer is facilitated by the favorable energy level alignment and the electronic coupling provided by the π-bridge. The electrons, now localized on the acceptor units, migrate through the polymer network to the catalyst surface. At the surface, these photogenerated electrons participate in the reduction of adsorbed protons (H^+^) to produce hydrogen gas (H_2_). Concurrently, the photogenerated holes remaining on the EDOT donor units are rapidly scavenged by the ascorbic acid (AA) sacrificial agent present in the reaction solution. The AA molecules are oxidized, consuming the holes and preventing them from recombining with the electrons. This hole scavenging process is crucial for maintaining charge separation and ensuring a continuous supply of electrons for hydrogen evolution. The overall result is a highly efficient photocatalytic cycle driven by the rational molecular design of the D-A-π-A copolymer, which integrates efficient light absorption, rapid exciton dissociation, efficient charge separation, and favorable surface reaction kinetics, leading to the outstanding hydrogen evolution performance observed for EDOT-BTDO-T.

## 3. Experimental Section

### 3.1. Materials

Analytical-grade chemical reagents were used as received without further purification, and deionized water was utilized in all experiments. 3,7-dibromodibenzob [d, b] dthiophene 5,5-dioxide, 3,7-bis(4,4,5,5-tetramethyl-1,3,2-dioxaborolan-2-yl) dibenzob [d, b] thiophene-5,5-dioxide, and 2,5-dibromo-3,4-ethylenedioxythiophene were prepared following previously reported procedures or obtained from commercial suppliers [59,60]. The specific preparation process can be found in the Supporting Information with the schematic diagram as shown in Scheme S1.

### 3.2. Synthesis of EDOT-BTDO-x Copolymers

A series of binary conjugated polymer photocatalysts, denoted as EDOT-BTDO-*x*, were prepared by Suzuki–Miyaura cross-coupling polymerization, with the schematic diagram as shown in Scheme S2. The variable x represents the molar feed ratio of the dibenzothiophene sulfone (BTDO) monomer to the 3,4-ethylenedioxythiophene (EDOT) monomer. A representative synthesis for EDOT-BTDO-5 is described as follows: 3,7-dibromodibenzob [d, b] dthiophene 5,5-dioxide (50 mg), 2,5-dibromo-3,4-ethylenedioxythiophene (20 mg), and 3,7-bis(4,4,5,5-tetramethyl-1,3,2-dioxaborolan-2-yl) dibenzob [d, b] dthiophene 5,5-dioxide (93.3 mg) were dispersed in 30 mL of N,N-dimethylformamide (DMF) in a 100 mL three-necked flask under ultrasonication for 25 min. Subsequently, an aqueous solution of sodium carbonate (2 mL, 2 M) and tetrakis(triphenylphosphine)palladium(0) (Pd(PPh_3_)_4_, 15 mg, 0.012 mmol) were added. The reaction mixture was heated to 140 °C and stirred under a nitrogen atmosphere for 48 h. After cooling to room temperature, the obtained precipitate was isolated by vacuum filtration, washed alternately with deionized water and methanol, and then dehydrated under vacuum. The resulting material was obtained as an orange powder [61]. As demonstrated in the Supporting Information, EDOT-BTDO-5 was identified as the optimal photocatalyst among the D-A type copolymers; therefore, the corresponding donor/acceptor feed ratio was adopted for subsequent investigations.

### 3.3. Synthesis of EDOT-BTDO-T Copolymer

**Scheme 1:**
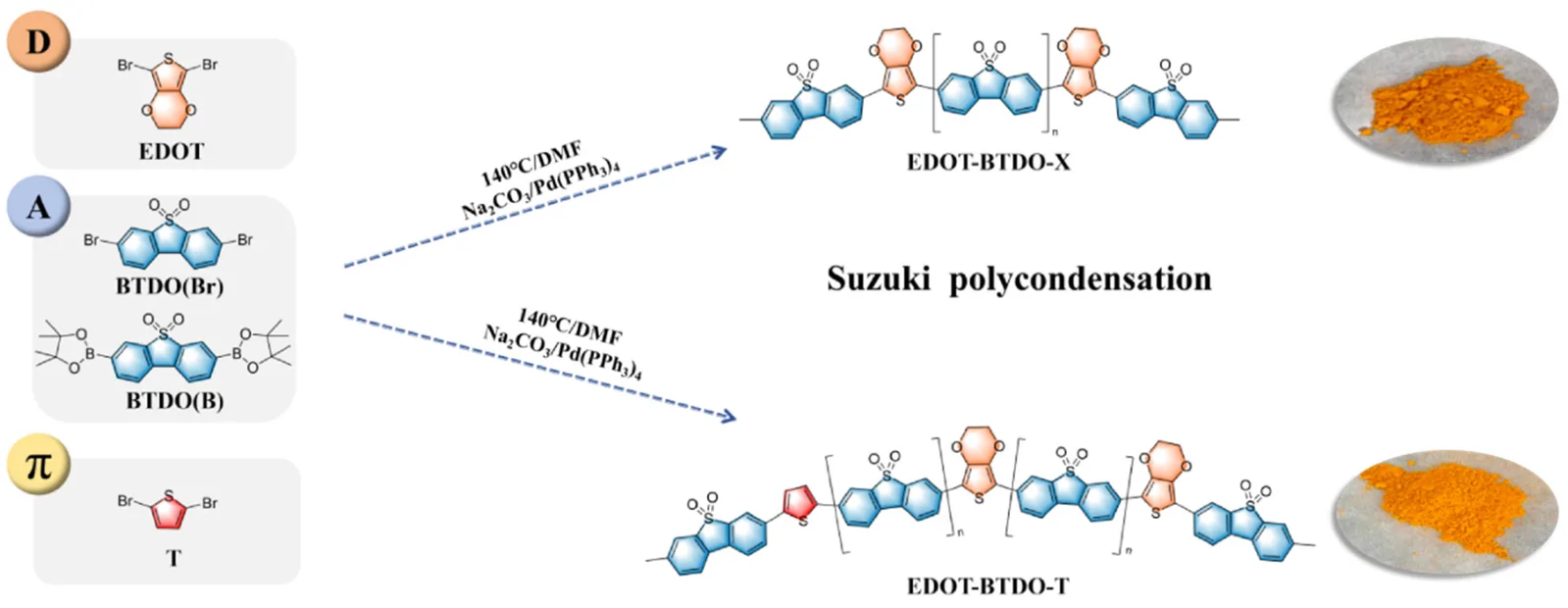
Synthesis of EDOT-BTDO-5 and EDOT-BTDO-T.

The ternary copolymer, designated EDOT-BTDO-T, was synthesized based on the optimized binary system (EDOT-BTDO-5) by introducing thiophene as a π-bridge. The synthesis followed a similar Suzuki polycondensation procedure. 3,7-bis(4,4,5,5-tetramethyl-1,3,2-dioxaborolan-2-yl) dibenzob [d, b] dthiophene 5,5-dioxide (93.5 mg), 3,7-dibromodibenzob [d, b] dthiophene (50 mg), 2,5-dibromo-3,4-ethylenedioxythiophene (15 mg), and 2,5-dibromothiophene (4.1 mg) were dispersed in a mixture of DMF and aqueous Na_2_CO_3_ solution. Pd(PPh_3_)_4_ (15 mg, 0.012 mmol) was introduced, and the mixture was subjected to ultrasonication for 25 min, followed by reflux at 140 °C under an argon atmosphere for 48 h. The product was collected by filtration, thoroughly the obtained solid was rinsed successively with methanol and deionized water, and subsequently dried under reduced pressure [38], and the images of the resulting copolymers are shown in Scheme 1.

### 3.4. Photocatalytic Hydrogen Evolution Measurements

Photocatalytic H_2_ production measurements were performed under visible light using a Labsolar-6A apparatus. Generally, 10 mg of the photocatalyst was dispersed in 5 mL of DMF and 95 mL of an aqueous ascorbic acid (AA) solution (1 M, pH adjusted to 3.5) as a sacrificial electron donor. The mixture was ultrasonicated and transferred to a 150 mL quartz reactor. The system was purged with nitrogen gas to eliminate dissolved oxygen. The reaction was carried out under visible-light irradiation (λ > 420 nm) using a 300 W Xe lamp with a cut-off filter. The evolved hydrogen was analyzed by gas chromatography (GC, Shimadzu GC-2014, Kyoto, Japan) at 30 min intervals for 4 h [61].

### 3.5. Characterization

The structure, photoelectrochemical characteristics, and photocatalytic performance of the synthesized EDOT-BTDO-5 and EDOT-BTDO-T, copolymers are characterized through FT-IR, solid-state ^13^C NMR, XRD, SEM, Uv-Vis absorption spectroscopy, Photoluminescence, Photocurrent Measurements, Mott-Schottky analysis, and Electrochemical Impedance Spectroscopy (EIS) techniques. DFT calculations, along with detailed experimental procedures and instrumentation, are described in the Supporting Information.

## 4. Conclusions

To summarize, we have successfully synthesized a series of D-A and D-A-π-A conjugated polymer photocatalysts based on a 3,4-ethylenedioxythiophene donor and a dibenzothiophene sulfone acceptor via Suzuki polycondensation. The optimized D-A binary copolymer, EDOT-BTDO-5, exhibited a notable HER of 87.5 mmol h^−1^ g^−1^ without any noble-metal cocatalyst. By further introducing a thiophene π-bridge to construct a D-A-π-A ternary copolymer, EDOT-BTDO-T, the photocatalytic performance was significantly enhanced to 103.45 mmol h^−1^ g^−1^, while maintaining excellent operational stability over 20 h. Comprehensive characterization and DFT calculations revealed that the D-A-π-A architecture promotes a more planar molecular conformation, extends π-conjugation, narrows the optical bandgap, and, most importantly, creates a strong intramolecular charge transfer pathway. This leads to efficient spatial separation of photogenerated charge carriers, suppressed charge recombination, and ultimately, superior photocatalytic H_2_ evolution. This work demonstrates a powerful molecular engineering strategy for designing high-performance, metal-free organic photocatalysts and offers valuable insights for the development of sustainable solar-to-fuel technologies.

## Figures and Tables

**Table 1:** Comparison of HER performance among organic conjugated photocatalysts reported in recent years and this work.

Material	Photocatalyst	HER (mmol g^−1^ h^−1^)	Wavelength Range	Sacrificial Agent	Cocatalyst	Journal/Year
inorganic semiconductors	meso-TiO_2_ NWs	35.70	full spectrum	-	Pt	[40]
COF/MCOF	Cu_3_-NDA MCOF	31.33	λ > 420 nm	TEA	-	[41]
inorganic semiconductors	Al-STO/Zn_x_Cd_1−x_S type-I heterojunction	21.15	λ > 420 nm	-	Pt	[42]
MOF	Ni single atoms @ UiO-66-NH_2_	17.61	Dark piezocatalysis	Methanol	Ni	[43]
MOF/COF	UPT-o-Cl	14.21	λ > 420 nm	-	Pt	[44]
COF	Ti_3_C_2_@In_2_O_3_@COF-3 ternary hybrid	12.90	λ > 420 nm	-	-	[45]
inorganic semiconductors	MoBT_x_ MBene/CdS	10.20	λ > 420 nm	lactic acid	-	[46]
inorganic semiconductors	CdS/Ti_3_C_2_-OH	5.42	λ > 420 nm	-	-	[47]
COF	TpBpyAn-Cu	5.33	λ > 420 nm	BIH	acetic acid	[48]
MOF	HP-UiO-66-NH_2_-Pyr	4.83	λ > 420 nm	-	Pt	[49]
organic polymers	CCN-3.5 crystalline g-C_3_N_4_	4.36	λ > 420 nm	TEOA	Pt	[50]
MOF	P-modified Co-Zn bimetallic MOF	4.10	λ > 420 nm	-	-	[51]
MOF	Ho-NH_2_-TPTC/CdS rare-earth MOF/CdS composite	3.80	λ > 420 nm	-	-	[52]
organic polymers	pyridine-based D-A type g-C_3_N_4_ copolymer	3.06	λ > 420 nm	-	Pt	[53]
organic polymers	CTF-Quin-OMe quinoline-based covalent triazine framework	1.45	λ > 420 nm	TEA	Pt	[54]

## Data Availability

The authors confirm that the data supporting the findings of this study are available within the article and as its Supplementary Materials.

## Supplementary Materials

The following supporting information can be downloaded at: https://www.mdpi.com/article/10.3390/molecules31173115/s1.
