# Supplementary material for: Thiophene π-Bridge Engineering for Boosting Photocatalytic H2 Evolution of Dioxythiophene-Based D-A-π-A Conjugated Polymers Without Extraneous Noble Metal Loading
Source: Molecules. 2026 Sep 5;31(17):3115. doi: 10.3390/molecules31173115 (PMC13567313; doi:10.3390/molecules31173115)
Supplement: Supplementary file 1 [file molecules-31-03115-s001.zip › molecules-4501266-supplementary.pdf]

Supporting Information

Thiophene π-Bridge Engineering for Boosting Photocatalytic H_2 Evolution of Dioxythiophene-Based D-A-π-A Conjugated Polymers Without Extraneous Noble Metal Loading

Guangsen Tian [1], Hongxi Zhao [1], Jinchen Zhang [1], Shaojia Song [2], Linfeng Zhang [1,3,*], Huadong Wu [1], Feng Wang [4], Jianding Li [5], Jia Guo [1,*] and Qun Yi [1,*]

[1] State Key Laboratory of Green and Efficient Development of Phosphorus Resources, Key Laboratory for Green Chemical Process of Ministry of Education, Hubei Key Laboratory of Novel Reactor and Green Chemical Technology, Engineering Research Center of Phosphorus Resources Development and Utilization of Ministry of Education, School of Chemical Engineering and Pharmacy, Wuhan Institute of Technology, Wuhan 430205, China; 15228972673@163.com (G.T.)

[2] College of Chemistry and Chemical Engineering, Taiyuan University of Technology, Taiyuan 030024, China

[3] School of Chemical Engineering and Technology, Tianjin University, Tianjin 300072, China

[4] School of Physical Education, Wuhan Business University, Wuhan 430056, China

[5] School of New Energy and Materials, Huzhou Normal University, Huzhou 313000, China

* Correspondence: lfzhang@wit.edu.cn (L.Z.); guojia@wit.edu.cn (J.G.); yq20210901@wit.edu.cn (Q.Y.)

Experimental:

Chemicals:

The deionized water used in the experiment comes from a pure water meter. The , Bis(pinacolato)diboron, Dibenzothiophene sulfone, sulfuric acid, N-Bromosuccinimide (NBS), Ethanol, Potassium acetate, N, N-Dimethylformamide (DMF), [1,1'-Bis(diphenylphosphino) ferrocene]dichloropalladium, Petroleum ether, Pd(dppf)Cl_2, Ethylacetate, Sodium carbonate, Tetrakis(triphenylphosphine)palladium[Pd(pph_3)$_4$], and Dichloromethane, were purchased from Aladdin Chemical Co Ltd. All chemicals are commercially available in an analytical grade, and no further purification is required for use.

Synthesis of dibenzothiophene sulfone

10.29 g of dibenzothiophene was dissolved in 100 mL of acetic acid solution. The mixture was heated to 100 °C and maintained at this temperature. Subsequently, 25 mL of hydrogen peroxide was slowly added dropwise to the system. The reaction was stirred continuously for 4 h, after which heating was stopped and the mixture was allowed to cool to room temperature. The reaction mixture was filtered under vacuum, and the collected filter cake was washed repeatedly with deionized water until neutral. Finally, the product was dried under vacuum to obtain colorless needle-like crystals.

Synthesis of 3,7-dibromodibenzothiophene sulfone

Under ice-bath conditions, 8.42 g of dibenzothiophene sulfone obtained from the previous step was dissolved in 150 mL of concentrated sulfuric acid. After complete dissolution, N-bromosuccinimide (NBS) was added slowly in batches. The reaction was stirred for an additional 6 h under ice-bath conditions. Upon completion, the mixture was slowly poured into

excess ice water, resulting in the precipitation of a white solid. The precipitate was collected by vacuum filtration, washed alternately with deionized water and anhydrous ethanol until neutral, and dried. The crude product was recrystallized from dichloromethane to afford white needle-like crystals.

Synthesis of 3,7-bis(4,4,5,5-tetramethyl-1,3,2-dioxaborolan-2-yl) dibenzothiophene 5, 5-dioxide

To a dry reaction vessel, 5.795 g of 3,7-dibromodibenzothiophene sulfone, 9.46 g of bis(pinacolato)diboron, and 8.76 g of potassium acetate were added, along with an appropriate amount of N, N-dimethylformamide (DMF) as solvent. The system was purged with argon to create an oxygen-free atmosphere, followed by the addition of 0.154 g of [1, 1'-bis(diphenylphosphino)ferrocene]palladium(II) dichloride ($Pd(dppf)Cl_2$) as catalyst. The reaction mixture was heated to 100 °C and stirred for 24 h. After the reaction, the product was extracted, the solvent was removed under reduced pressure, and the residue was purified by column chromatography to obtain a white solid product, and the synthesis route of the precursors are shown in **Scheme S1**.

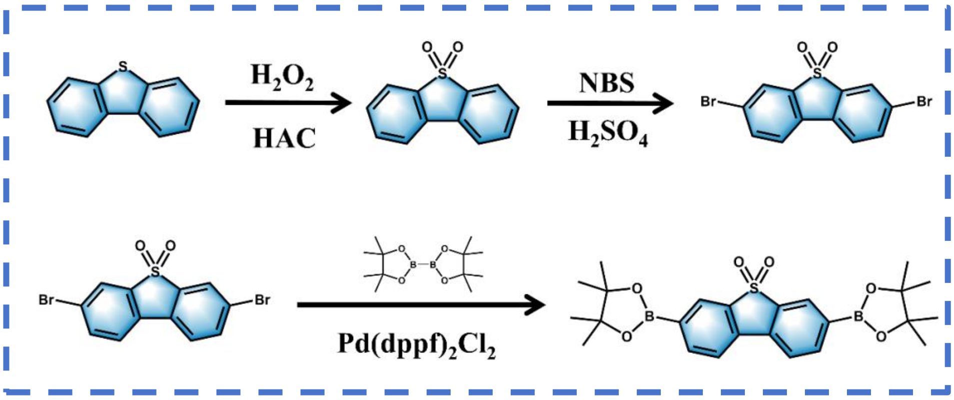

Scheme S1 Synthesis route of the catalyst precursors

Preparation process of the copolymer

A series of binary conjugated polymer photocatalysts, denoted as EDOT-BTDO-*x*, were prepared by Suzuki–Miyaura cross-coupling polymerization. The variable *x* represents the molar feed ratio of the dibenzothiophene sulfone (BTDO) monomer to the 3, 4-ethylenedioxythiophene (EDOT) monomer. In this work, EDOT-BTDO-5, EDOT-BTDO-3, EDOT-BTDO-7, and EDOT-BTDO-9 were synthesized. Photoelectrochemical characterization of these copolymers was performed, and the results are presented in the following figures (**Scheme S2**).

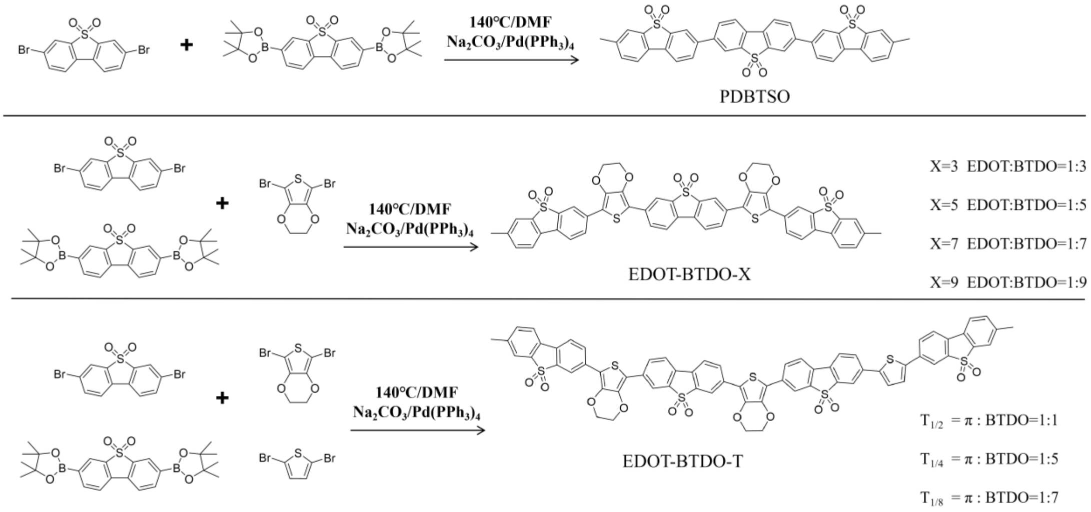

Scheme S2 Synthesis route of PDBTSO、EDOT-BTDO-*x*、EDOT-BTDO-*T*

Characterization

Powder X-ray diffraction measurements were made by X-ray diffractometer (XRD, Bruker D8 Advance, Germany) at a scanning angle of 5-90°. The chemical bonds of the samples were analyzed by Fourier infrared spectroscopy (FT-IR, NICOLET 5700, USA). The surface functional groups of the samples were observed by X-ray photoelectron spectroscopy (XPS, Escalab 250XI, ThermoFisher, USA). The composition of the sample was further verified by solid-state nuclear magnetism (SSNMR, Bruker Avance Neo 400 WB, Germany). The specific surface area and nitrogen adsorption-desorption isotherm of the samples were analyzed by BET. The temperature of differential thermal analyzer (Q1000DSC+LNCS+FACS Q600SDT) was 30-800 °C, and the

heating rate was 10 °C min^{-1}. The morphology of the samples was characterized by scanning electron microscopy (SEM, JSM-6700F). The content of Pd in copolymer was determined by inductively coupled plasma-mass spectrometry (ICP-MS, Agilent 5110). The Uv-Vis absorption spectra of the samples were calculated by ultraviolet visible spectrophotometer (UV, SPC UV-3600, Jp), The test range is from 200 nm to 800 nm. The samples were analyzed by photoluminescence spectroscopy (PL) using a fluorescence spectrometer (F-4600 FL, Japan) with an excitation wavelength of 350nm and a scanning range of 200-800 nm. The photocurrent, electrochemical impedance spectroscopy (EIS) and Mott-Schottky curves were measured by CHI 600 Electrochemical workstation (Shanghai, China). The self-made three-electrode quartz battery was immersed in the electrolyte solution with $K_3Fe(CN)_6$, $K_4Fe(CN)_6$ and KCl concentrations of 0.1 M, and the above-mentioned workstation and 300 W Xenon lamp (pl-sxe 300) were used. The and Mott-Schottky curve were studied with 0.1 M Na_2SO_4 as electrolyte solution. Photocurrent measurements were performed using 0.1 M Na_2SO_4 solution as the electrolyte. A 300 W xenon lamp (Model: PL-SXE 300) was employed as the light source, with an on-off cycling mode at intervals of 20 seconds. The theoretical calculations were performed via the Gaussian 16 suite of package. The structure of the studied molecules were fully optimized at the B3LYP-D3BJ/6-311G(d,p) level of theory. The vibrational frequencies of the optimized structures were carried out at the same level. The structure was characterized as a local energy minimum on the potential energy surface by verifying that all the vibrational frequencies were real. The highest occupied orbital (HOMO) and lowest unoccupied orbital (LUMO) orbitals were calculated and analyzed using Multiwfn software, and the isosurfaces were visualized using the Visual Molecular Dynamics (VMD) program. The electrostatic potential (ESP) was computed and analyzed using

the Multiwfn software, and the corresponding isosurfaces were visualized with the Visual Molecular Dynamics (VMD) program. The external quantum efficiency of hydrogen evolution was measured by 10 mg polymer with 420, 475, 520, and 600 nm monochromatic light with band-pass filter, and the intensities were 68.78, 75.48, 91.08 and 112.42 mW cm^{-2}, respectively. The irradiation area was controlled to 3.14 cm^2. The AQY produced by H_2 under monochromatic light is shown below.

$$\mathrm{AQY(\%)}=\frac{2\times\text{Number of evolved } H_2 \text{ molecules}}{\text{Number of incident photons}}=\frac{2\times M\times N_A}{S\times P\times t\times\frac{\lambda}{h\times c}}$$

Where, M is the amount of hydrogen molecules (mol), N_A is avogadro constant (6.022×10^{23} mol^{-1}), h is the Planck constant (6.626×10^{-34} J·s), c is the speed of light (3×10^{8} m s^{-1}), S is the irradiation area (cm^2), P is the intensity of irradiation light (W cm^{-2}), t is the photoreaction time (s), λ is the wavelength of the monochromatic light (m).

Figures:

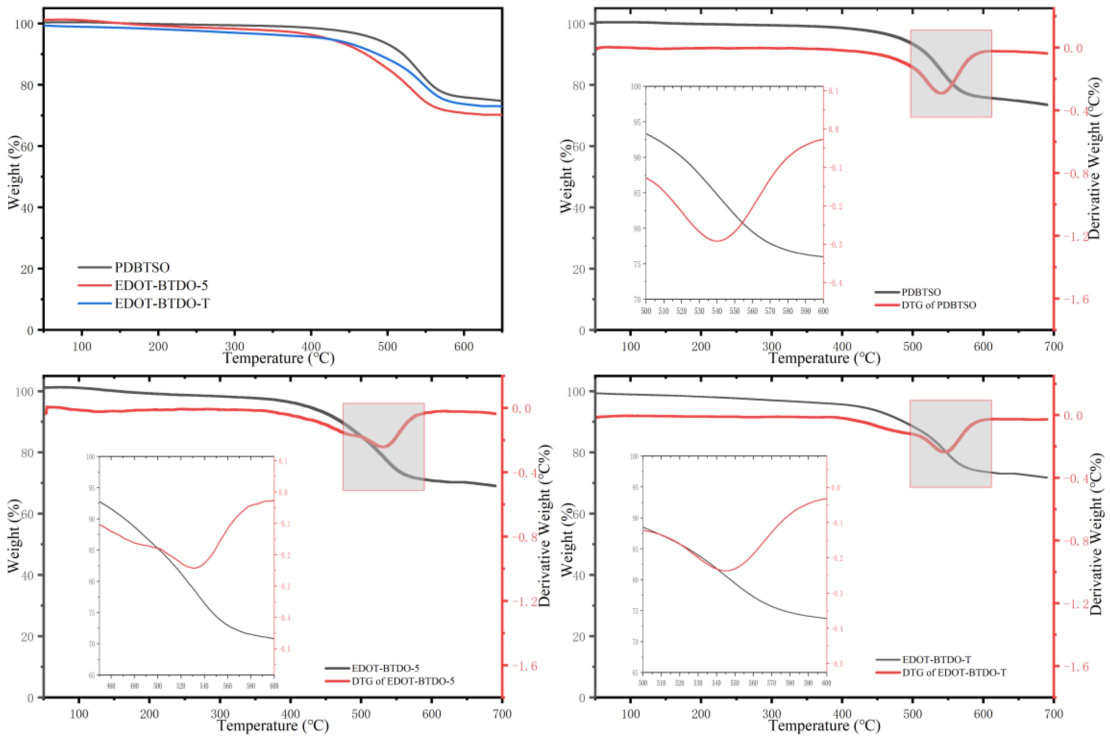

Figure S1 TGA curves of the copolymers

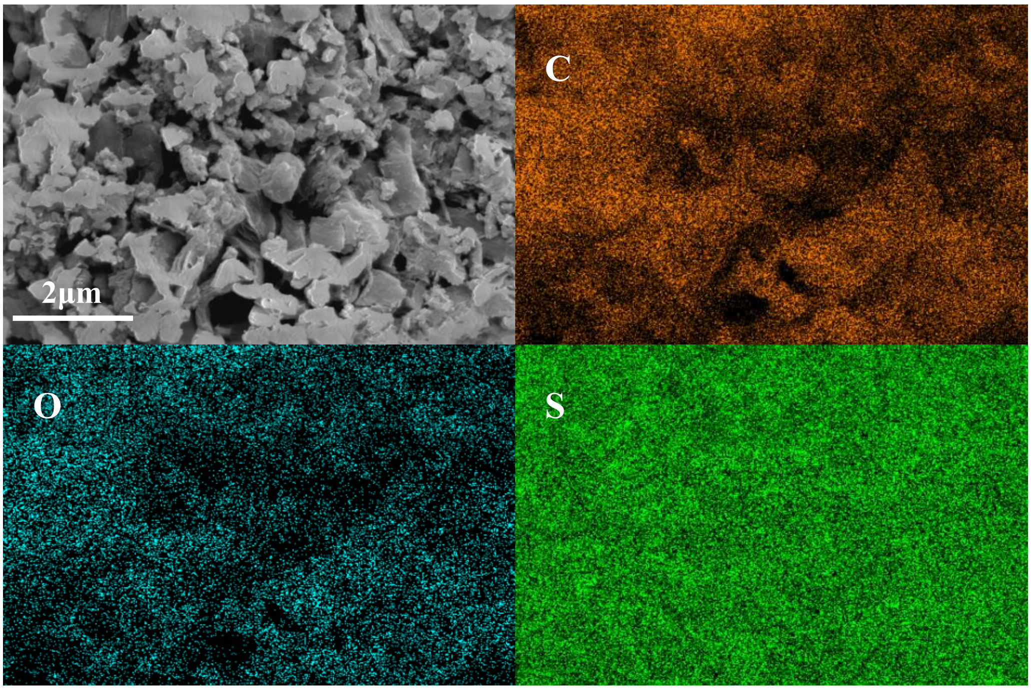

Figure S2 SEM image and EDX mapping of EDOT-BTDO-T

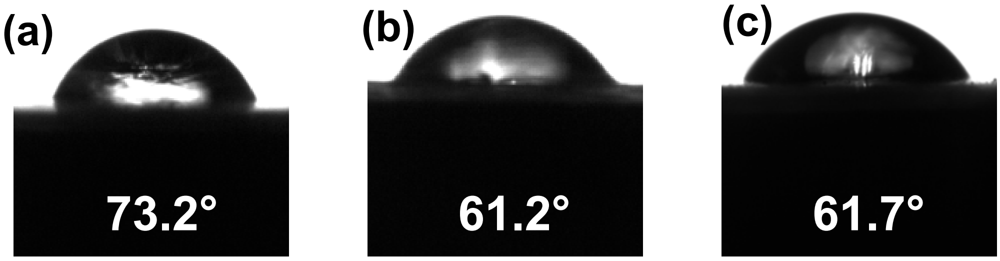

Figure S3 Water contact angle of PDBTSO, EDOT-BTDO-5 and EDOT-BTDO-T

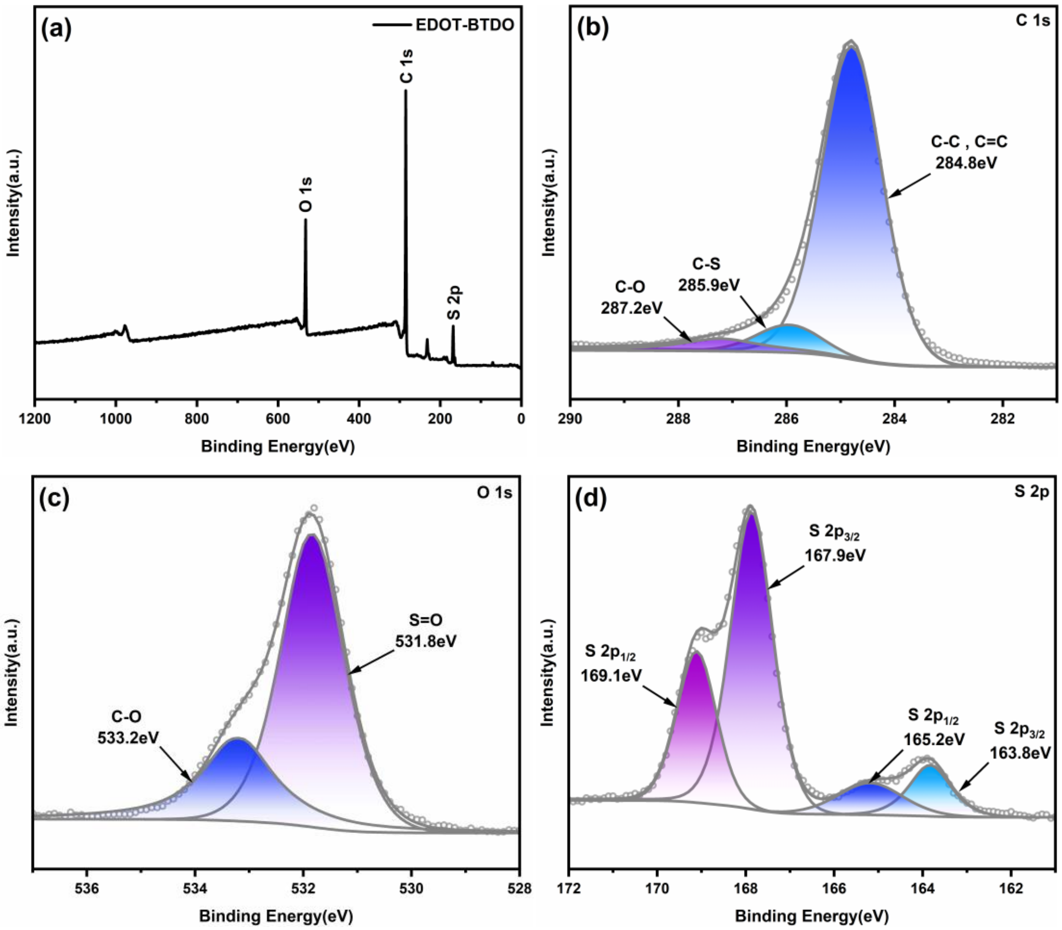

Figure S4 (a) XPS survey spectrum of EDOT-BTDO-5; High resolution XPS spectrum of EDOT-BTDO-5; (b) C 1*s*; (c) S 2*p* and (d) O 1*s*

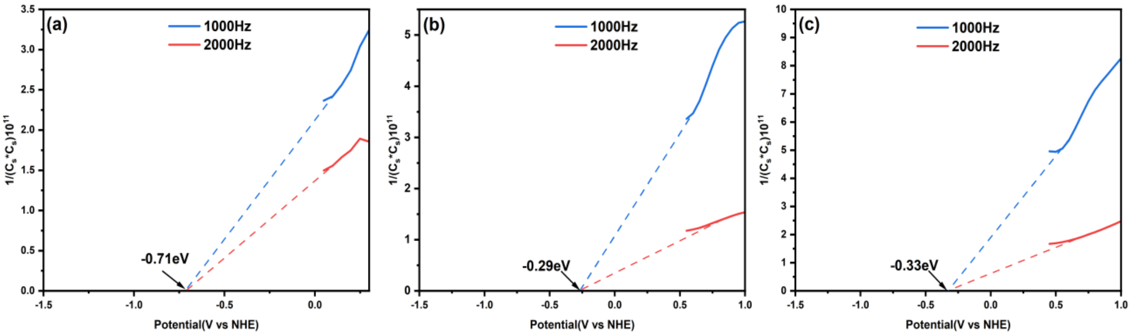

Figure S5 Mott-Schottky plots of the polymers

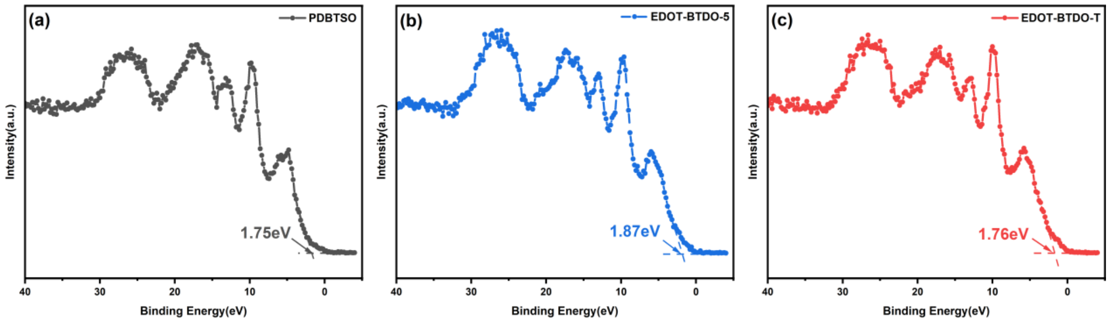

Figure S6 Valence band spectra of (a) PDBTSO, (b) EDOT-BTDO-5, and (c) EDOT-BTDO-T

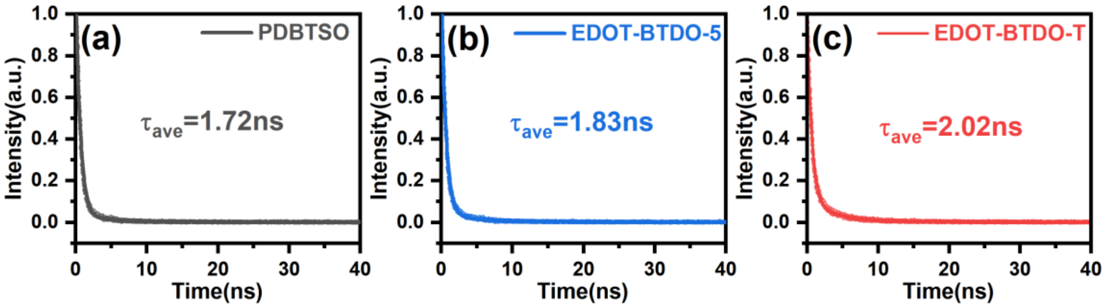

Figure S7 Fluorescence decay curves of PDBTSO, EDOT-BTDO-5 and EDOT-BTDO-T

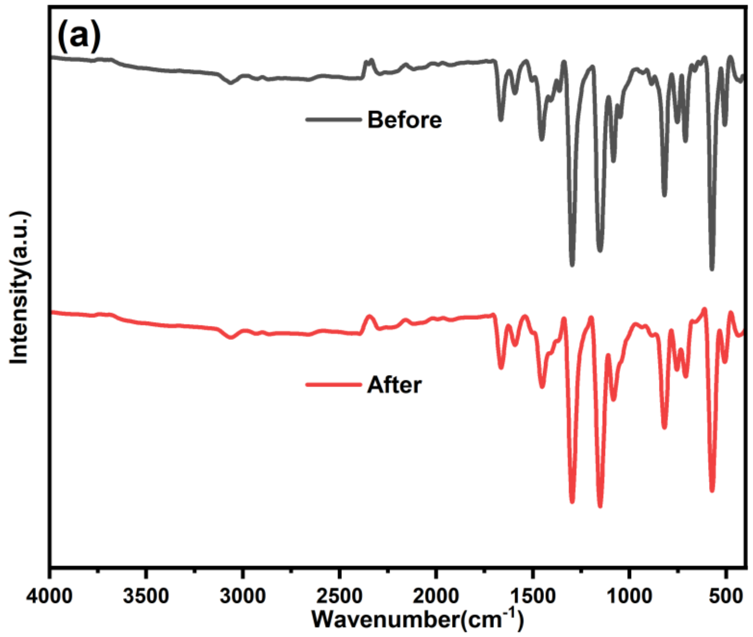

Figure S8 FT-IR spectra of EDOT-BTDO-T before and after photocatalytic reaction in DMF/water mixed solution

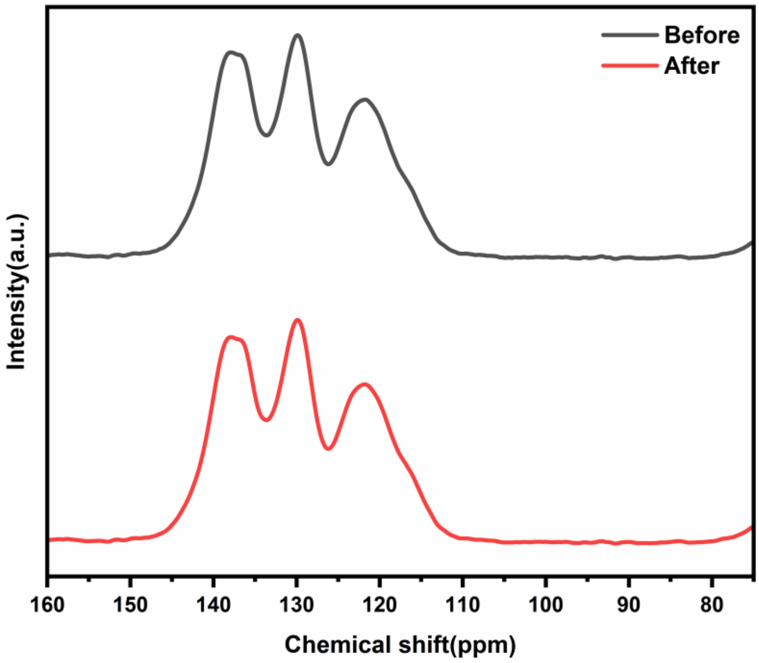

Figure S9 solid-state ^{13}C NMR spectra of EDOT-BTDO-T before and after photocatalytic reaction in DMF/water mixed solution

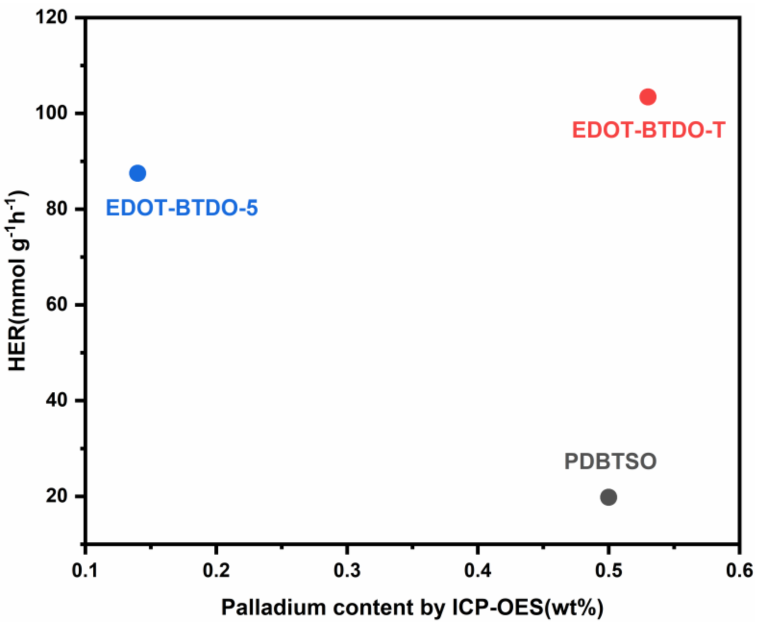

Figure S10 Palladium content and hydrogen evolution rate of the three samples

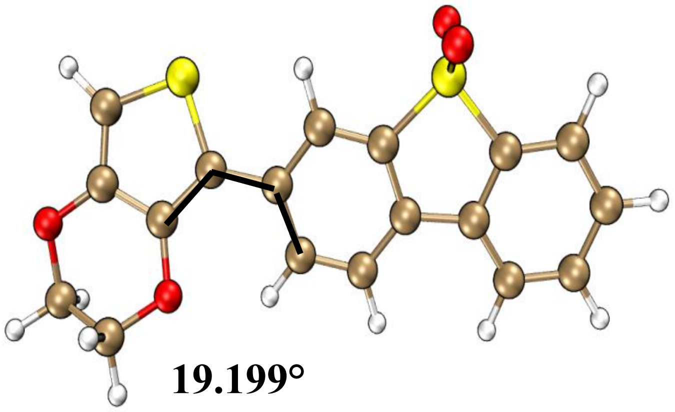

Figure S11 Molecular structure and dihedral angle diagrams of EDOT-BTDO-5 polymers

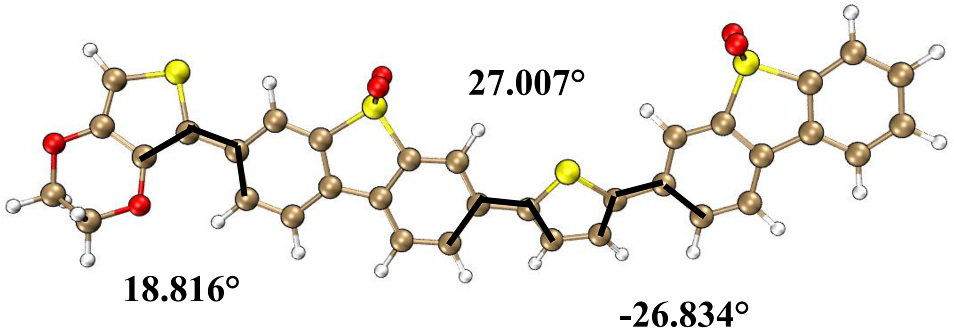

Figure S12 Molecular structure and dihedral angle diagrams of EDOT-BTDO-T polymers

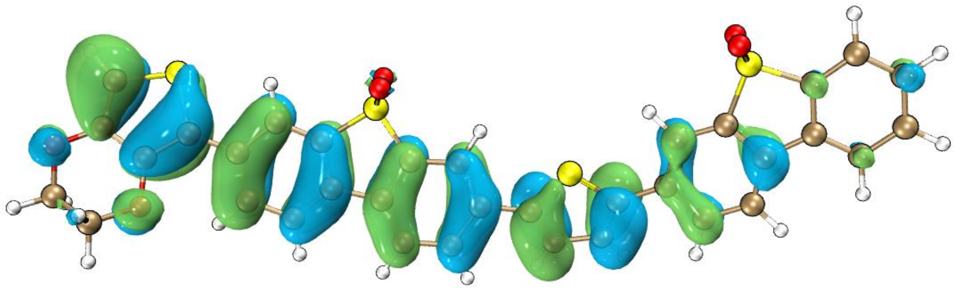

Figure S13 The HOMO orbital of EDOT-BTDO-T polymers

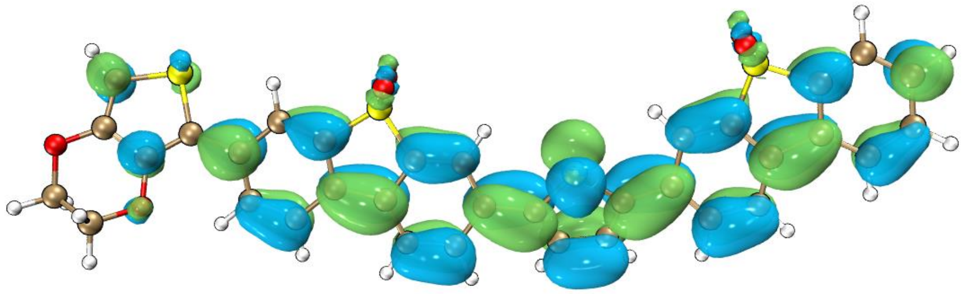

Figure S14 The LUMO orbital of EDOT-BTDO-T polymers

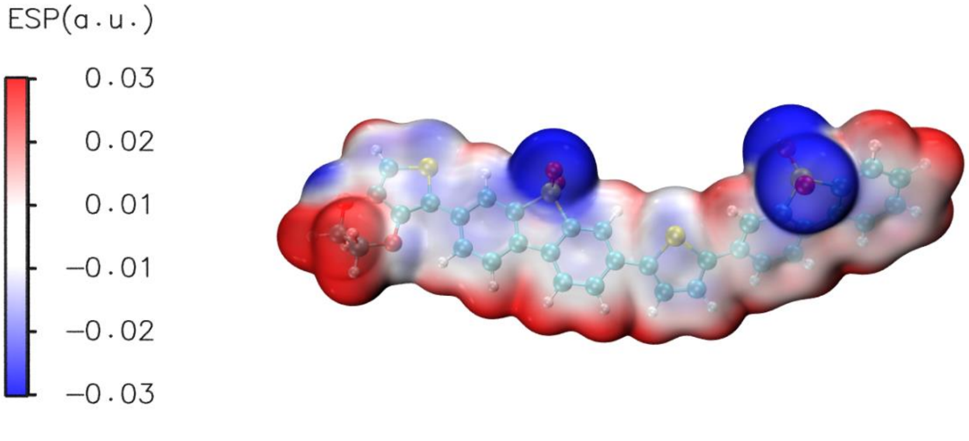

Figure S15 Electrostatic potential (ESP) mapping of EDOT-BTDO-T
